# Overexpression of *OsERF48* causes regulation of *OsCML16*, a calmodulin‐like protein gene that enhances root growth and drought tolerance

**DOI:** 10.1111/pbi.12716

**Published:** 2017-03-27

**Authors:** Harin Jung, Pil Joong Chung, Su‐Hyun Park, Mark Christian Felipe Reveche Redillas, Youn Shic Kim, Joo‐Won Suh, Ju‐Kon Kim

**Affiliations:** ^1^ Graduate School of International Agricultural Technology and Crop Biotechnology Institute/GreenBio Science and Technology Seoul National University Pyeongchang Korea; ^2^ Center for Nutraceutical and Pharmaceutical Materials Division of Bioscience and Bioinformatics Myongji University Yongin Gyeonggi Korea; ^3^ Present address: Laboratory of Plant Molecular Biology Rockefeller University New York NY USA

**Keywords:** *OsERF48*, drought tolerance, root growth, co‐regulatory network, calmodulin‐like protein

## Abstract

The AP2/ERF family is a plant‐specific transcription factor family whose members have been associated with various developmental processes and stress tolerance. Here, we functionally characterized the drought‐inducible *OsERF48*, a group Ib member of the rice ERF family with four conserved motifs, CMI‐1, ‐2, ‐3 and ‐4. A transactivation assay in yeast revealed that the C‐terminal CMI‐1 motif was essential for OsERF48 transcriptional activity. When *OsERF48* was overexpressed in an either a root‐specific (*ROX*^*O*^ ^*s*^ ^*ERF*^ ^*48*^) or whole‐body (*OX*^*O*^ ^*s*^ ^*ERF*^ ^*48*^) manner, transgenic plants showed a longer and denser root phenotype compared to the nontransgenic (NT) controls. When plants were grown on a 40% polyethylene glycol‐infused medium under *in vitro* drought conditions, *ROX*^*O*^ ^*s*^ ^*ERF*^ ^*48*^ plants showed a more vigorous root growth than *OX*^*O*^ ^*s*^ ^*ERF*^ ^*48*^ and NT plants. In addition, the *ROX*^*O*^ ^*s*^ ^*ERF*^ ^*48*^ plants exhibited higher grain yield than *OX*^*O*^ ^*s*^ ^*ERF*^ ^*48*^ and NT plants under field‐drought conditions. We constructed a putative *OsERF48* regulatory network by cross‐referencing *ROX*^*O*^ ^*s*^ ^*ERF*^ ^*48*^ root‐specific RNA‐seq data with a co‐expression network database, from which we inferred the involvement of 20 drought‐related genes in *OsERF48*‐mediated responses. These included genes annotated as being involved in stress signalling, carbohydrate metabolism, cell‐wall proteins and drought responses. They included, *OsCML16*, a key gene in calcium signalling during abiotic stress, which was shown to be a direct target of OsERF48 by chromatin immunoprecipitation‐qPCR analysis and a transient protoplast expression assay. Our results demonstrated that OsERF48 regulates *OsCML16*, a calmodulin‐like protein gene that enhances root growth and drought tolerance.

## Introduction

Plants dehydrate during limited water conditions, whereupon they close their stomata to avoid further water loss, while experiencing a stress‐induced reduction in growth. Roots are the first organs to sense drought conditions, and their regulation of water uptake is believed to be an important mechanism for managing restricted water availability (Matsuo *et al*., 2009). A robust root system is therefore both valuable for enhancing plant growth and integral in water stress responses (Uga *et al*., 2013). Root development is under complex cellular and metabolic control, and many of the associated processes are known to be regulated by specific transcription factors (TFs), including members of the APETALA2/ethylene‐responsive element binding factor (AP2/ERF), MYB, bZIP and NAC families (reviewed by Nakashima *et al*., 2014).

The AP2/ERF superfamily is one of the largest plant‐specific TF families. Proteins in this family contain a single AP2/ERF domain and are classified into ten subgroups (I–X), each with unique conserved motifs (Nakano *et al*., 2006). Two motifs have been functionally characterized: one from group VIII (CMVIII‐1), which is identical to an ERF‐associated amphiphilic repression (EAR) motif (Ohta *et al*., 2001) and the CMIX‐1 domain from *Arabidopsis thaliana* group IX, which is a transcriptional activation domain with a unique ‘EDLL’ motif (Tiwari *et al*., 2012). The other conserved motifs have yet to be characterized. AP2/ERF family members have been associated with root developmental processes and stress tolerance. *A. thaliana AP2/EREBP PUCH1* and poplar (*Populus trichocarpa*) *PtaERF003* promote lateral root formation (Hirota *et al*., 2007; Trupiano *et al*., 2013), while rice (*Oryza sativa*) *ERF3/AP37* is essential for crown root development (Zhao *et al*., 2015) and, when overexpressed, significantly increases grain yield under field‐drought conditions (Oh *et al*., 2009). It was proposed that a particularly well‐developed root system in the *ERF3/AP37* overexpressing plants was responsible for the increased grain yield (Zhao *et al*., 2015). In addition, overexpression of the *A. thaliana HARDY* gene in rice improved water‐use efficiency by increasing root biomass under drought conditions (Karaba *et al*., 2007). Recently, it was reported that overexpression of *OsERF71* (Lee *et al*., 2016a) and *HIGHER YIELD RICE*/*OsERF137* (Ambavaram *et al*., 2014) in rice resulted in vigorous root growth that improved grain yield under drought conditions. Finally, overexpression of other *AP2/ERF* TFs has been found to confer drought tolerance by elevating the levels of osmoprotectants, including proline and soluble sugars (Quan *et al*., 2010; Wang *et al*., 2015; Zhang *et al*., 2010).

In addition to TFs, Ca^2+^ acts as a core signal transducer and regulator in many plant adaptations to environmental conditions, and elevated levels of cytosolic calcium concentration are central to many stress responses (Zeng *et al*., 2015). Major transduction routes of Ca^2+^ signalling involve the calcium‐binding proteins, calmodulin (CaM) and CaM‐like proteins (CML), calcium‐dependent protein kinases (CDPKs) and calcineurin‐B‐like proteins (CBL) (Reddy *et al*., 2011). CMLs are multifunctional proteins that interact with a broad range of Ca^2+^ binding downstream targets by directly or indirectly binding to their promoters, in combination with calmodulin‐binding proteins (CBPs). These interactions mediate the regulation of various target proteins, such as protein kinases/phosphatases, transcription factors, metabolic enzymes, ion channels and structural proteins (reviewed by *Reddy et al*., 2011; Zeng *et al*., 2015). There is considerable evidence that CMLs play a critical role in Ca^2+^ signalling during plant adaptations to abiotic stress in tomato (*Solanum lycopersicum*) (Munir *et al*., 2016), *A. thaliana* (Magnan *et al*., 2008; Park *et al*., 2010) and rice (Xu *et al*., 2011). However, the molecular mechanisms by which CMLs regulate stress responses remain unknown.

In the present study, we investigated the roles of *OsERF48*, a rice gene encoding an ERF TF, by overexpressing it in transgenic rice plants in either a root‐specific or whole‐body expression manner. Root‐specific overexpressors showed improved drought tolerance by a promotion of root growth than whole‐body overexpressors or nontransgenic (NT) control plants. By cross‐referencing RNA‐seq data with a co‐expression database, we constructed a putative regulatory network of *OsERF48* in which *OsCML16*, a calmodulin‐like protein, plays a central role. Our results suggest that OsERF48 regulates *OsCML16*, which in turn enhances root growth and drought tolerance.

## Results

### OsERF48 is a drought‐inducible transcriptional activator

**Figure 1:**
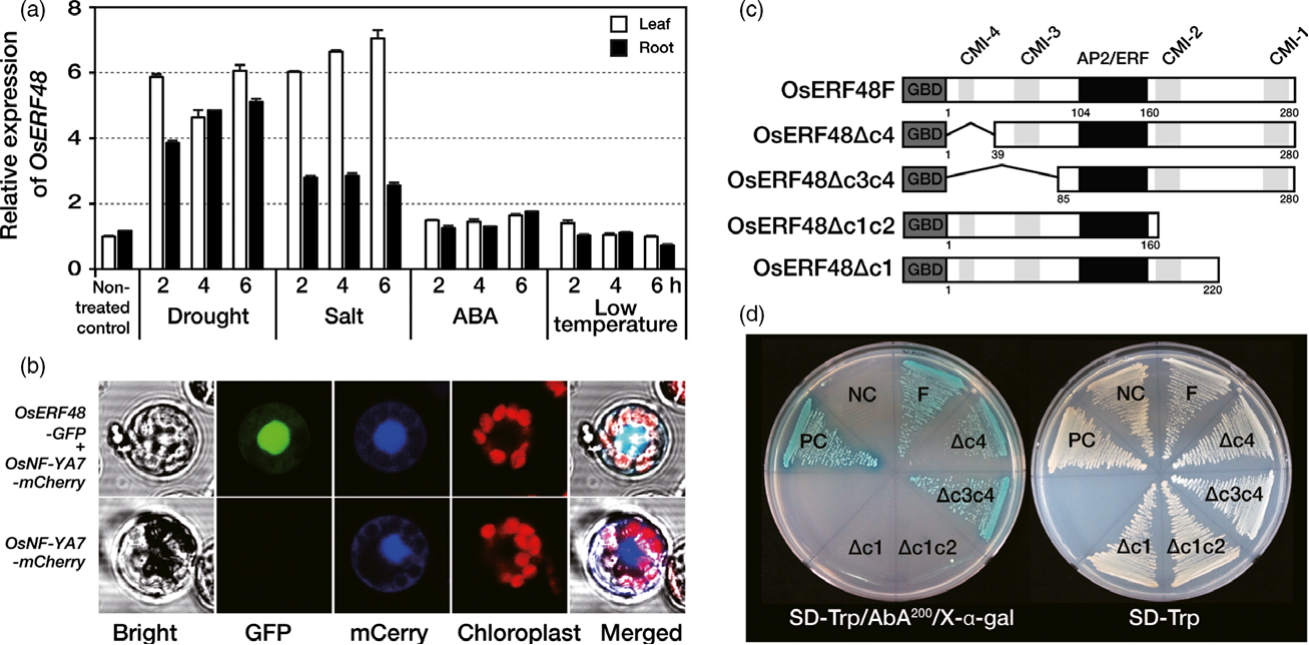
OsERF48 is a nuclear protein with transcriptional activation. (a) Relative expression of *OsERF48* in response to abiotic stresses. Two‐week‐old seedlings were exposed to air‐drying (drought), 400 mm NaCl (salt), 100 μm abscisic acid (ABA) at 28 °C and at 4 °C (low temperature) for the indicated time points. *OsUbi1* expression was used as an internal control. Values are the means ± SD (standard deviation) of three independent experiments. (b) Subcellular localization of OsERF48 in rice protoplasts. Protoplasts were transiently co‐transformed with *OsERF48‐GFP* and the nuclear localization control *OsNF‐YA7‐mCherry*. Fluorescence was observed using a confocal microscope. (c–d) Transactivation activity of OsERF48 using a yeast system. (c) Schematic structure of OsERF48 full length and deletion mutants. (d) Transformed yeast cells harbouring the indicated constructs on SD/‐Trp and SD/‐Trp/AbA^200^/X‐α‐gal. NC, negative control (*pGBKT7*); PC, positive control (*pGBKT7‐53 + pGADT7‐RecT*); AP2/ERF, AP2/ERF domain, CMI, conserved motif of group I, GBD, GAL4 DNA‐binding domain.

*OsERF48* (Os08g0408500) transcript levels increased upon exposure of 2‐week‐old rice seedlings to drought and high‐salt conditions, but not upon exposure to the hormone abscisic acid (ABA) and low temperature (Figure 1a). To verify that OsERF48 was localized in the nucleus, which would be expected for a transcription factor, a construct with the full‐length *OsERF48* translationally fused to *green fluorescent protein* (*GFP*) was transformed into rice protoplasts, together with a gene encoding the nuclear localized reporter *OsNF‐YA7‐mCherry* as a positive control (Lee *et al*., 2015). GFP and mCherry fluorescence co‐localized in the nucleus of rice protoplasts (Figure 1b), indicating nuclear localization of OsERF48.

To confirm the transcriptional activity of OsERF48, the GAL4 DNA‐binding domain (DBD) was fused to a full‐length or deletion mutants of OsERF48 (Figure S1) and reporter genes expressed in yeast under the control of the GAL4 target‐binding site. Overexpression of the full‐length protein (OsERF48F) or either of two N‐terminal deletion mutants (OsERF48Δc4 and OsERF48Δc3c4) induced expression of the reporter genes, demonstrating their transcriptional activity (Figure 1c, d). However, C‐terminal deletions of CMI‐1 and CMI‐2 (OsERF48Δc1c2), or CMI‐1 only (OsERF48Δc1), resulted in no induction of the reporter genes (Figure 1c, d), suggesting that CMI‐1 is important for the transcriptional activity of OsERF48.

### Overexpression of *OsERF48* in rice confers drought tolerance at the vegetative stage

**Figure 2:**
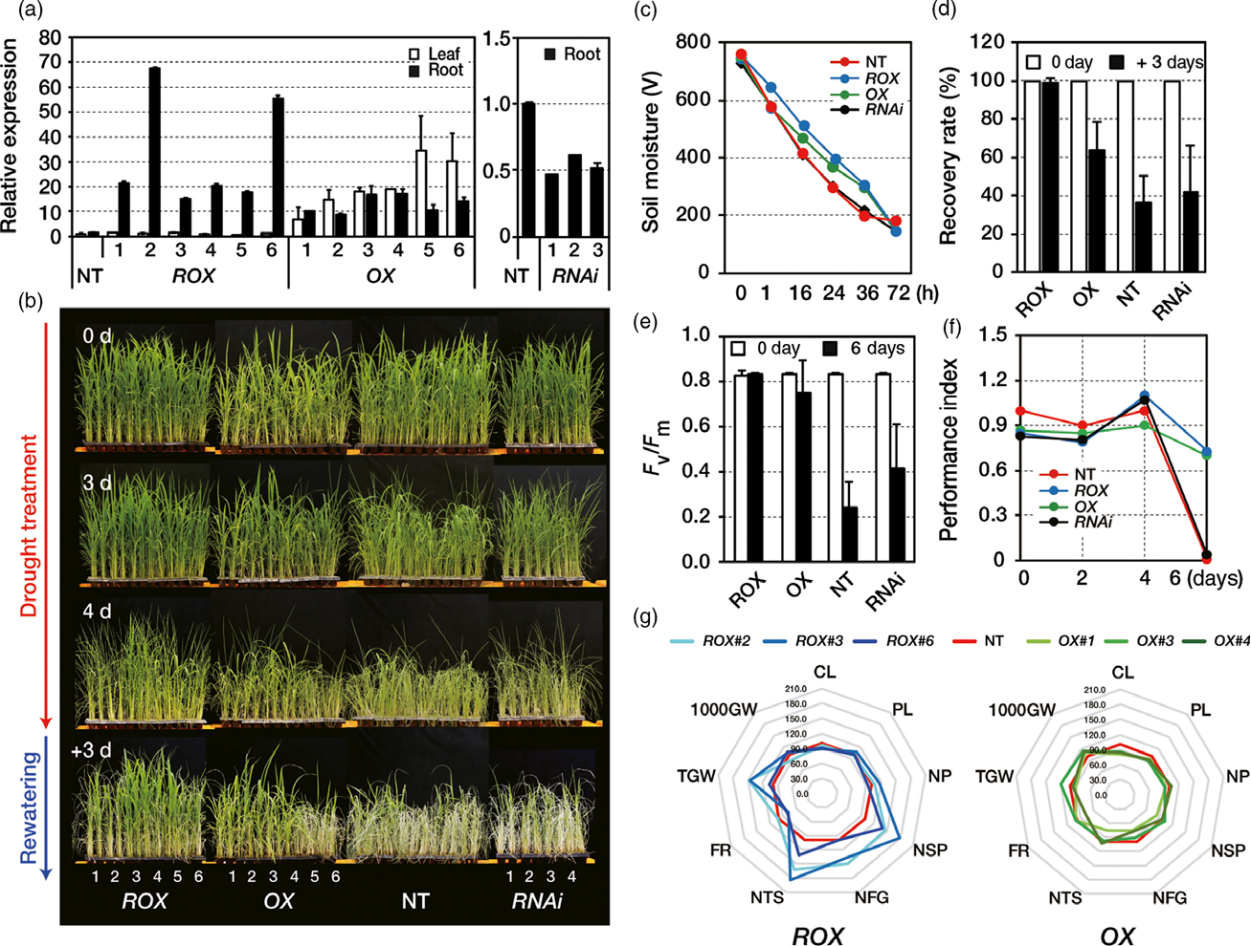
Drought tolerance of *OsERF48* overexpressing plants. (a) Relative expression of *OsERF48* transcripts in transgenic and nontransgenic (NT) plants. Two‐week‐old roots or leaves from transgenic plants were used for the analysis. *OsUbi1* expression was used as an internal control. Values are the means** **± SD of three independent experiments. (b–d) All plants were grown in soil for 1 month under well‐watered conditions and were then exposed to drought stress for 4 days, followed by re‐watering for 3 days in the greenhouse. (b) Drought tolerance of transgenic and NT plants. (c) Soil moisture in the pots exposed to drought treatment at the indicated time points. Values are the means** **± SD (*n* = 20). (d) Recovery rate scored 7 days after re‐watering. Values are the means** **± SD (*n* = 30). (e, f) Determination of the photosynthetic viability of transgenic and NT plants under drought conditions. All plants were grown in soil for 2 months under well‐watered conditions and then exposed to drought stress for 6 days. At the indicated time point after exposure to drought stress, the chlorophyll fluorescence (*F*_*v*_ */F*_*m*_) (e) and performance index (f) of transgenic and NT plants were measured. Each data point represents the mean** **±**** SD (*n* = 30 points per independent lines of each genotypes). (g) Agronomic traits of transgenic and NT plants under drought conditions. The spider plot represents the agronomic traits by the percentage of the mean values (*n* = 18), listed in Table 1. Mean measurements from the NT control were assigned a 100% reference value. CL, culm length; PL, panicle length; NP, number of panicle; NSP, number of spikelet per panicle; NFG, number of filled grain; NTS, number of total spikelet; FR, filling rate; TGW, total grain weight; 1000GW, 1000 grain weight. *ROX*,*ROX*^*O*^ ^*s*^ ^*ERF*^ ^*48*^; *OX*,*OX*^*O*^ ^*s*^ ^*ERF*^ ^*48*^.

To investigate the biological function of *OsERF48*, we generated three types of transgenic rice plants, in the *Ilmi* cultivar: one with *r*oot‐specific *o*vere*x*pression (*ROX* ^*OsERF48*^); the second with whole‐body *o*vere*x*pression (*OX* ^*OsERF48*^); and the third with RNA‐interference (*RNAi* ^*OsERF48*^)‐mediated suppression of the *OsERF48* gene (Figure S2a, b). We chose single‐copy T_3_ homozygous transgenic lines for subsequent analysis (Figure S3) and showed that the *OsERF48* transcript levels were elevated in both leaves and roots of *OX* ^*OsERF48*^ and only in roots of *ROX* ^*OsERF48*^ plants, while they were decreased by 50% compared to nontransgenic control (NT) plants in *RNAi* ^*OsERF48*^ roots (Figure 2a).

To perform a drought tolerance test, 1‐month‐old transgenic and NT plants were exposed to drought by withholding water for several days. Soil moisture showed a constant decrease over this period, indicating that the stress was uniformly applied to the plants (Figure 2c). Visual drought‐associated symptoms, such as leaf rolling and wilting, occurred earlier in NT and *RNAi* ^*OsERF48*^ plants than in the *OsERF48* overexpressing plants (Figure 2b). Notably, all plants of the *ROX* ^*OsERF48*^ lines showed far fewer such symptoms than the other plants after 4 days of drought exposure, and they also had the highest recovery rate (99%) after re‐watering. Two of six *OX* ^*OsERF48*^ lines, as well as all the *RNAi* ^*OsERF48*^ and NT plants exhibited severe drought symptoms, indicative of a much lower recovery rate than the *ROX* ^*OsERF48*^ plants after re‐watering (Figure 2d). To verify the drought tolerance, we selected 3 independent transgenic lines harbouring each construct (#2, 3 and 6 for *ROX* ^*OsERF48*^; #1, 3 and 4 for *OX* ^*OsERF48*^; # 1, 2 and 4 for *RNAi* ^*OsERF48*^) and determined their *F*_*v*_ */F*_*m*_ values and performance index, which represent two different indicators of photochemical efficiency, under drought conditions. Leaves of the *ROX* ^*OsERF48*^ and the *OX* ^*OsERF48*^ plants remained more viable with higher *F*_*v*_ */F*_*m*_ values (Figure S4a, b) and performance index values after 6 days of drought treatment than the NT and *RNAi* ^*OsERF48*^ plants (Figure 2e, f), indicating a higher drought tolerance in the overexpression plants than in the knock‐down (RNAi) and NT controls.

### *ROX* ^*OsERF48*^ lines have increased grain yield under drought conditions

Since crop productivity is closely associated with drought stress, we evaluated yield components of the transgenic plants under normal and field‐drought conditions. *OsERF48* overexpressing plants exhibited several growth defects, such as reduced plant height, panicle and spikelet number, which collectively contributed to a lower total grain weight under normal growth conditions (Table 1). However, under field‐drought conditions, the number of total spikelets (NTS) and total grain weight (TGW) of the *ROX* ^*OsERF48*^ plants were 33%–85% and 6%–47% greater, respectively, while those of the *OX* ^*OsERF48*^ plants were similar to the NT plants (Table 1; Figure 2g). These results suggest that the *ROX* ^*OsERF48*^ plants showed enhanced drought tolerance during the reproductive stage of growth hence higher grain yield under field‐drought conditions.

**Table 1:** Agronomic traits of transgenic rice plants grown under normal and drought conditions

Genotype	Culm length (cm)	Panicle length (cm)	No. of panicle/hill	No. of spikelet/panicle	No. of total spikelet/hill	Filling rate (%)	Total grain weight (g)	1000 grain weight (g)
Normal condition								
NT	69.4	20.3	16.6	116.3	1935.5	91.5	46.8	26.6
*ROX‐2*	58.7**	21.2	10.8**	120.3	1283.1**	75.5**	26.2**	29.2
%Δ	−15.4	4.4	−35.3	3.4	−33.7	−17.4	44.1	10.0
*ROX‐3*	63.7**	21.4	13.0**	123.3	1575.9**	87.2**	38.0**	28.0*
%Δ	−8.2	5.7	−21.8	6.0	−18.6	−4.7	−18.8	5.5
*ROX‐6*	63.5**	19.5	11.4**	126.4**	1444.4**	76.6**	26.9**	25.3
%Δ	−8.4	−3.6	−31.1	8.6	−25.4	−16.3	−42.5	−4.7
*OX‐1*	64.4**	19.3	13.8**	104.2	1387.3**	85.9**	33.2**	28.1
%Δ	−7.1	−4.8	−17.2	−10.4	−28.3	6.1	−29.0	6.0
*OX‐3*	60.6**	18.8	16.8	100.1**	1657.2	82.3**	36.1**	26.7
%Δ	−12.7	−7.5	0.8	−13.9	−14.4	−10.0	−22.8	0.6
*OX‐4*	60.7**	18.4	15.0	87.0**	1307.4**	80.6**	28.3**	27.0
%Δ	−12.4	−9.2	−9.5	−25.2	−32.5	−11.9	−39.5	1.7
Drought condition								
NT	56.4	17.9	11.5	67.0	759.9	64.7	12.1	27.1
*ROX‐2*	54.9	17.8	12.4	98.0**	1224.8**	65.5	17.7**	23.9
%Δ	−2.8	−0.6	7.3	46.3	61.2	1.2	47.0	−11.7
*ROX‐3*	49.7**	19.3	13.4	120.0**	1404.0	49.5	17.6	28.6
%Δ	−11.9	8.1	16.2	79.2	84.8	−23.5	46.1	5.9
*ROX‐6*	51.4**	17.9	10.6	92.8**	1007.1	51.9	12.8	29.3
%Δ	−8.9	0.1	−7.7	38.5	32.5	−19.9	6.2	8.3
*OX‐1*	45.4**	16.6	10.7	56.3*	571.7**	65.5	10.7	29.5
%Δ	−19.4	−7.0	−7.5	−15.9	−24.8	1.3	−11.0	9.2
*OX‐3*	48.1**	15.7	10.5	70.0	740.0	65.5	14.3	30.9
%Δ	−14.6	−12.0	−9.3	4.4	−2.6	1.2	18.8	14.2
*OX‐4*	47.9**	16.3	12.1	65.5	785.3	50.7*	11.3	30.1
%Δ	−15.0	−9.0	4.8	−2.3	3.3	−21.7	−6.0	11.2

Each data values represents the mean (*n* = 30, normal conditions; *n* = 18, drought conditions) for transgenic and NT control plants. Single (*P < 0.05) and two asterisks (**P < 0.01) represent significant differences by the Student's *t*‐test between the transgenic and NT plants.

### *ROX* ^*OsERF48*^ lines have enhanced root growth under drought conditions

**Figure 3:**
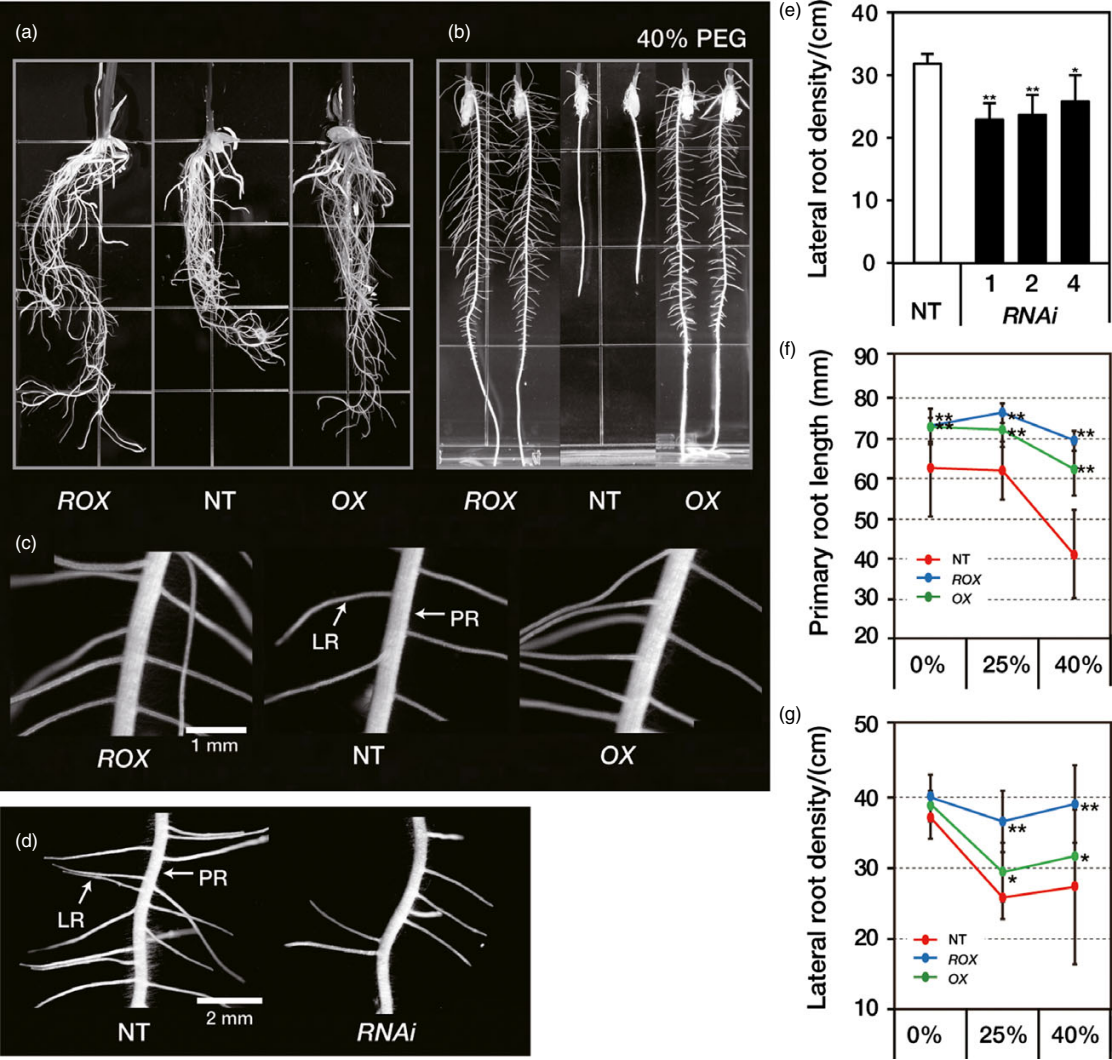
*OsERF48* overexpressing lines have vigorous root growth. (a–c) Comparison of 6‐day‐old seedlings roots of *OsERF48* overexpression and nontransgenic (NT) plants. (a) Root morphology of plants grown on normal growth media. (b) Root growth of plants grown on a 40% polyethylene glycol (PEG)‐infused media for *in vitro* drought conditions. (c) Lateral root morphology of plants grown on normal growth media (scale bar, 1 mm). (d) Lateral root morphology of *RNAi* ^*Os*^ ^*ERF*^ ^*48*^ and NT plants (scale bar, 2 mm). (e) Lateral root density of *RNAi* ^*Os*^ ^*ERF*^ ^*48*^ and NT plants. (f) Primary root length of the *OsERF48* overexpressing and NT plants grown on the PEG‐infused media. (g) Lateral root density of the *OsERF48* overexpressing and NT plants grown on the PEG‐infused media. Lateral root density was measured within a 3‐ to 4.5‐cm region from the root tip. Each data point represents the mean** **±**** SD (*n* = 15 plants per independent lines of each genotypes). A single (*P < 0.05) and two asterisks (**P < 0.01) represent significant differences, as determined by the student's *t*‐test between the transgenic and NT plants. PR, primary root; LR, lateral root; *ROX*,*ROX*^*O*^ ^*s*^ ^*ERF*^ ^*48*^; *OX*,*OX*^*O*^ ^*s*^ ^*ERF*^ ^*48*^; *RNAi*,*RNAi* ^*Os*^ ^*ERF*^ ^*48*^.

Under normal growth conditions, primary roots of *ROX* ^*OsERF48*^ and *OX* ^*OsERF48*^ plants were 19%, longer than NT roots, resulting in a deeper root growth (Figures 3a, S5a‐c). In addition, the *ROX* ^*OsERF48*^ and *OX* ^*OsERF48*^ roots had a higher lateral root density by 43.8% and 16.4%, respectively, than the NT plants (Figures 3c, S5d). In contrast, the primary root length of the *RNAi* ^*OsERF48*^ plants was similar to that of the NT plants, and the lateral root density was 28% lower (Figure 3d, e). The root‐to‐shoot (R/S) ratio of the *ROX* ^*OsERF48*^ and *OX* ^*OsERF48*^ plants was 65% and 64% higher, respectively, than the NT plants (Table 2), and this correlated with increased root dry weight values in the *ROX* ^*OsERF48*^ and *OX* ^*OsERF48*^ plants, which were approximately twice that of the NT plants (Table 2). We concluded that overexpression of *OsERF48* caused longer and denser root growth, resulting in a more vigorous root growth phenotype with higher R/S ratio.

**Table 2:** Root and shoot dry weights in *OsERF48* transgenic and nontransgenic (NT) plants

Genotype	Shoot		Root		R/S (%)
	Dry weight	Δ%	Dry weight	Δ%	
NT	49.85 ± 1.75		10.5 ± 1.63		0.21 ± 0.03
*ROX*					
2	60.13 ± 3.23*	20.6	20.90 ± 1.45**	98.6	0.35 ± 0.03**
3	59.13 ± 4.06*	18.6	20.77 ± 1.47**	97.3	0.35 ± 0.04**
6	61.47 ± 3.50*	23.3	21.20 ± 2.96*	101.4	0.34 ± 0.07
Average	60.24 ± 3.50	23.3	20.96 ± 2.96	101.4	0.35 ± 0.07
*OX*					
1	51.33 ± 4.26	3.0	21.40 ± 1.47**	103.3	0.42 ± 0.06**
3	58.03 ± 4.91	16.4	19.70 ± 2.88*	87.2	0.34 ± 0.03**
4	49.70 ± 2.79	−0.3	16.30 ± 4.19	54.9	0.33 ± 0.11
Average	53.87 ± 2.79	8.1	18.00 ± 4.19	71.0	0.33 ± 0.11

Mean ± SD followed by a single (*P < 0.05) or two asterisks (**P < 0.01) represent significant differences as determined by the Student's *t*‐test between the transgenic and nontransgenic (NT) plants. *ROX*,* ROX* ^*OsERF48*^; *OX*,* OX* ^*OsERF48*^.

To observe the root phenotype under drought conditions, seedlings were grown in PEG‐infused media (0%, 25% and 40% PEG). Figure 3b shows that under severe drought conditions (40% PEG), roots of the *ROX* ^*OsERF48*^ plants were denser and the lateral roots were longer than those of the *OX* ^*OsERF48*^ plants. Root growth of the NT and *OX* ^*OsERF48*^ plants in 40% PEG‐infused media was severely retarded in terms of primary root length (by 35% and 19%, respectively) and lateral root density (both by 27%). Under the same drought conditions, the *ROX* ^*OsERF48*^ plants maintained their root growth with far less of a reduction in primary root length (5.3%) and lateral root density (2.3%) than the other plants. Thus, under drought conditions, the *ROX* ^*OsERF48*^ plants had a more vigorous root phenotype than the NT and *OX* ^*OsERF48*^ plants, which is consistent with enhanced drought tolerance.

### Construction of an *OsERF48* transcriptional co‐regulatory network

To identify *OsERF48* target genes, we performed a RNA‐seq analysis on roots of two independent *ROX* ^*OsERF48*^ lines with high *OsERF48* transcript levels (*ROX#2* and *ROX#6*) together with nontransgenic plants grown under normal conditions. A total of 200 differentially expressed genes (DEGs) with significant changes in transcript abundance were found, of which 159 and 41 were up‐ and down‐regulated in *ROX* ^*OsERF48*^ roots, respectively, compared to NT roots (Figure S6a, b). To verify the transcriptome profile, we analysed the expression of 18 putatively up‐regulated genes by qRT‐PCR, using *ROX#2* and *ROX#6* root samples, which gave a similar pattern of gene expression to the RNA‐seq data (Figure S6c).

**Figure 4:**
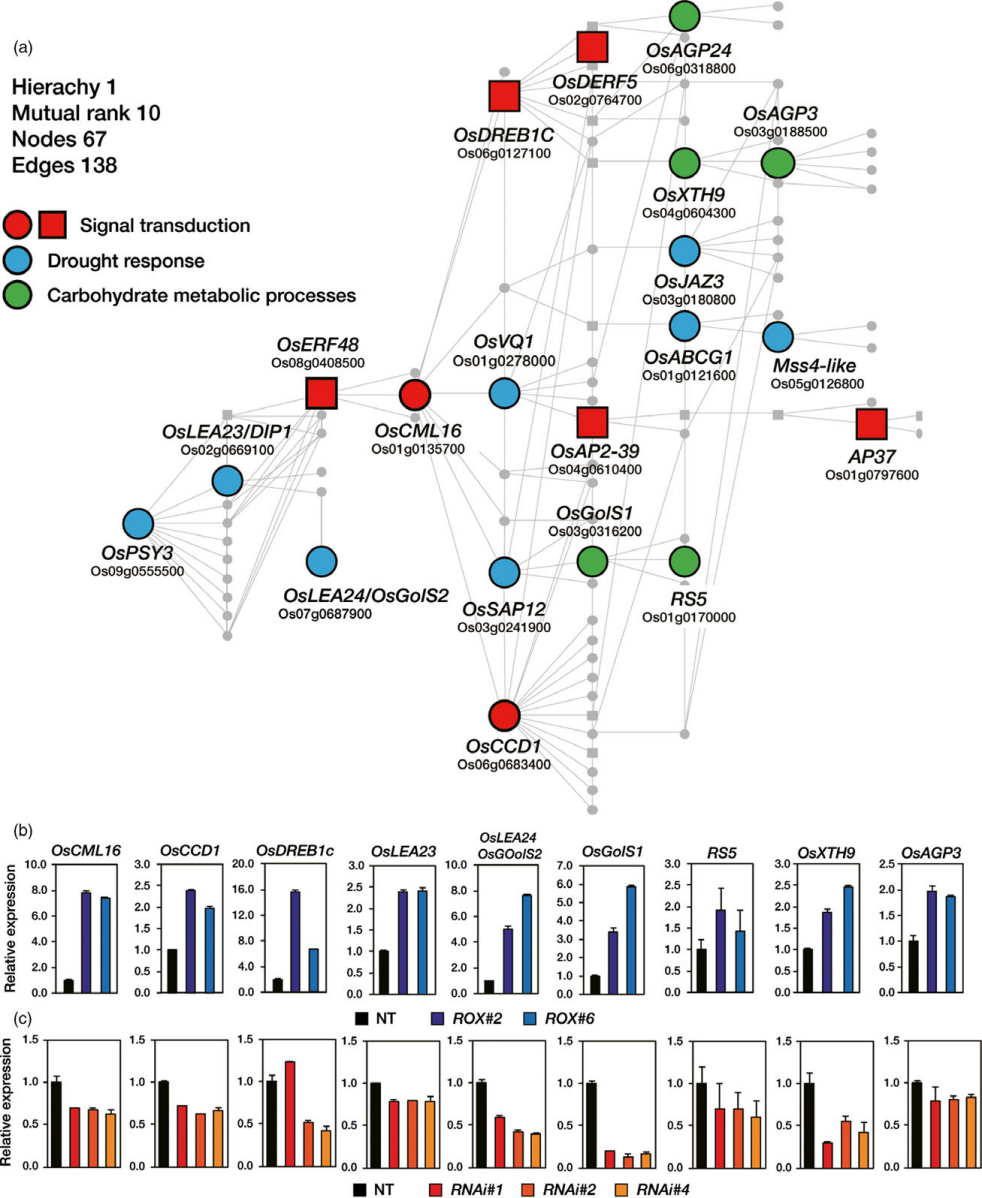
*OsERF48* transcriptional co‐regulatory network. (a) *OsERF48* and its co‐expressed genes were selected from the differentially expressed genes (DEGs) in *ROX*^*O*^ ^*s*^ ^*ERF*^ ^*48*^ roots and used as guide input in the RiceFREND web tool to construct this network. The network comprises 67 genes (nodes), of which 20 were present amongst the *ROX*^*O*^ ^*s*^ ^*ERF*^ ^*48*^ root DEGs. Big nodes represent *OsERF48* plus the 19 identified *ROX*^*O*^ ^*s*^ ^*ERF*^ ^*48*^ root DEGs. All other genes are depicted as small grey circles. Square nodes represent transcription factors. All genes in this network are listed in Table S2. (b–c) qRT‐PCR analysis showing the transcript levels of 9 genes from the network in 2‐week‐old *ROX*^*O*^ ^*s*^ ^*ERF*^ ^*48*^ (b) and *RNAi* ^*Os*^ ^*ERF*^ ^*48*^ (c) roots. *OsUbi1* expression was used as internal control. Values are the means** **±**** SD of three independent experiments. *ROX*,*ROX*^*O*^ ^*s*^ ^*ERF*^ ^*48*^; *OX*,*OX*^*O*^ ^*s*^ ^*ERF*^ ^*48*^; *RNAi*,*RNAi* ^*Os*^ ^*ERF*^ ^*48*^.

In order to construct a co‐regulatory network for *OsERF48*, we selected 56 genes (Table S1) from the 200 DEGs identified in the *ROX* ^*OsERF48*^ roots by filtering with the following criteria: (i) genes that were up‐regulated in the *ROX* ^*OsERF48*^ roots; (ii) genes that were up‐regulated in drought‐treated rice roots available in a public database (Kawahara *et al*., 2016); and (iii) genes that were annotated in RAB‐DB (http://rapdb.dna.affrc.go.jp/) and NCBI (http://www.ncbi.nlm.nih.gov) (Figure S6d). We then generated a co‐expression matrix of those 56 genes based on the ‘single‐gene guide’ approach of the RiceFREND (http://ricefrend.dna.affrc.go.jp/) web tool and sorted the genes with high co‐expression frequency (Figure S7). The *OsERF48* co‐regulatory network was constructed by applying the 20 selected genes from the co‐expression matrix using the ‘multi‐gene guide’ approach in RiceFREND (Figure 4a; Table S2). The 20 genes, including *OsERF48*, were classified into three groups based on GO terms: ‘signal transduction’, ‘carbohydrate metabolism’ and ‘stress response’ (Table 3). The expression levels of the nine genes were verified by qRT‐PCR analysis of 2‐week‐old *ROX* ^*OsERF48*^ and *RNAi* ^*OsERF48*^ root samples (Figure 4b, c). Genes associated with ‘signal transduction’–*CALMODULIN‐LIKE PROTEIN 16* (*OsCML16*), a small calcium‐binding protein, *C‐TERMINAL CENTRIN‐LIKE DOMAIN 1* (*OsCCD1*) and *DEHYDRATION RESPONSE ELEMENT‐BINDING PROTEIN 1c* (*OsDREB1c*), ‘drought response’–*LATE EMBRYOGENESIS ABUNDANT PROTEIN 23*/*DEHYDRATION INDUCIBLE PROEIN 1* (*OsLEA23/DIP1*) and *OsLEA24/GALACTINOL SYNTHASE 2* (*OsLEA24/OsGolS2*), and ‘carbohydrate metabolism’–*OsGolS1*,* RAFFINOSE SYNTHASE 5* (*RS5*), *XYLOGLUCAN ENDOTRANSGLUCOSYLASE‐HYDROLASE 9* (*OsXTH9*) and *ARABINOGALACTAN PROTEIN 3* (*OsAGP3*) were observed to be up‐regulated in *ROX* ^*OsERF48*^ roots and down‐regulated in the *RNAi* ^*OsERF48*^ roots, supporting the validity of the constructed network.

**Table 3:** Expression level of the 20 genes in the *OsERF48* co−regulatory network

Description	ID	ROX/WT.fc	ROX/WT.pval	Dro/con*	Co−exp. freq.^†^	References	Calmodulin−binding motif (in 2−kb promoter)^‡^
Signal transduction							
OsCML16	Os01g0135700	4.9	0.003	6.00	18	Yu *et al*. (2013)^4^	−1181, −1061, −901
OsCCD1	Os06g0683400	2.8	0.044	3.74	13	Jing *et al*. (2016)^1^	−445, −399
OsERF048	Os08g0408500	6.0	0.000	2.53	7	In this study Oh *et al*. (2009)^1^	−808, −640
OsDREB1c	Os06g0127100	5.2	0.006	5.21	20	Ito *et al*. (2006)^2^	−1889, −1811, −1803, −1791,− 284, −175
OsDERF5	Os02g0764700	4.2	0.006	4.61	13	Oh *et al*. (2009)^1^	−1992, −1923, −1918, −1865,−1859, −1758, −56
OsAP2−39	Os04g0610400	3.1	0.028	4.02	13	Oh *et al*. (2009)^1^	−1350, −1308, −1268, −1155, −1117, −1098, −1057, −894, −779
OsAP37/OsERF3	Os01g0797600	2.2	0.025	2.86	8	Oh *et al*. (2009)^1^; Zhao *et al*. (2015)^1^	−1474, −1431, −1418, −1381, −1362, −1309, −1193, −1085, −31
Carbohydrate metabolic processes							
OsXTH9	Os04g0604300	3.1	0.000	3.26	7	Yang *et al*. (2006)^2^; Osato *et al*. (2006)^3^	−1263, −1253, −1234, −821, −791, −648, −622
OsAGP24	Os06g0318800	2.6	0.010	3.35	7	Gong *et al*. (2012)^3^	−492, −303
OsAGP3	Os03g0188500	2.4	0.011	2.01	3	Gong *et al*. (2012)^3^	−835, −750, −333
RS5	Os01g0170000	2.8	0.000	2.69	4	Wu *et al*. (2009)^4^	−1479
OsGolS1	Os03g0316200	3.6	0.000	4.83	7	Wu *et al*. (2009)^4^; Taji *et al*. (2002)^3^; Shimosaka and Ozawa (2015)^3^; Zhuo *et al*. (2013)^3^	
OsGolS2/ OsLEA24	Os07g0687900	3.3	0.002	5.30	9	He *et al*. (2017)^4^; Wu *et al*. (2009)^4^	−1703, −1700
Response to stimulus							
DIP1/ OsLEA23	Os02g0669100	2.2	0.004	2.39	13	Jung *et al*. (2015)^4^; *He et al*. (2017)^4^	−1859, −1795, −1460, −791, −785, −431, −295, −198
OsPSY3	Os09g0555500	2.0	0.000	6.89	13	Welsch *et al*. (2008)^2^	−1943, −1906, −333, −273, −213, −177, −155, −94, −83, −4
OsSAP12	Os03g0241900	2.6	0.011	2.94	11	Merewitz *et al*. (2011)^1^	−1812, −1480
OsJAZ3	Os03g0180800	3.9	0.006	5.28	7	Ye *et al*. (2009)^1^; Seo *et al*. (2011)^4^	−958
OsVQ1	Os01g0278000	2.2	0.021	3.34	12	Li *et al*. (2014)^2^	−1398, −1368, −1316, −1302, −1264, −1252, −1250
OsABCG1	Os01g0121600	2.2	0.000	3.55	9	Matsuda *et al*. (2012)^2^	
Mss4	Os05g0126800	2.5	0.001	2.40	7		−667, −581, −489, −256, −63

*Fold‐change (log2) between roots of drought‐treated/not‐treated controls in wild‐type rice plant (Kawahara *et al*., 2016).

^†^Represents a co‐expression frequency between the indicated gene and the 56 candidate genes identified in *ROX* ^*OsERF48*^ roots based on co‐expression matrix shown in Figure S7.

^‡^Represents presence of calmodulin‐binding motifs from the translation start site (+1) determined using PlantPAN (similar score = 1). Superscripts 1–4 on references reports observations of the following: (1) Its overexpression has drought tolerance or enhanced root growth. (2) Induced by abiotic stresses. (3) Orthologous gene related with abiotic stress. (4) Up‐regulated in transgenic plants having abiotic stress tolerance.

### OsERF48 directly binds to the promoter of *OsCML16*, a key gene in calcium signalling in response to abiotic stress

**Figure 5:**
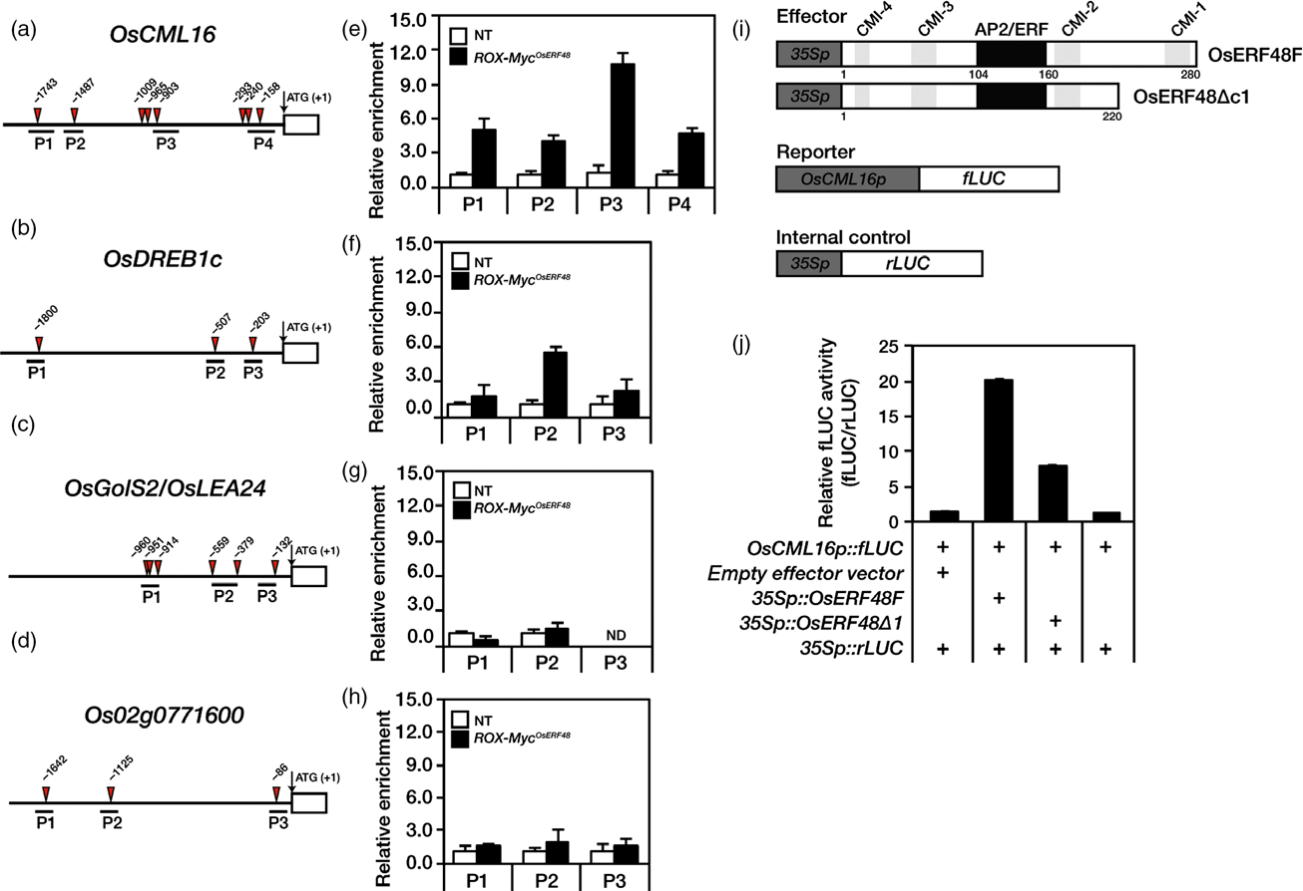
Chromatin immunoprecipitation (ChIP)‐qPCR and transient protoplast expression assays showing that OsERF48 interacts with the *OsCML16* promoter. (a–h) Two‐week‐old *ROX‐Myc* ^*Os*^ ^*ERF*^ ^*48*^ and nontransgenic (NT) roots were used in the ChIP‐qPCR experiments with an anti‐myc antibody. (a–d) Promoter region showing ChIP‐qPCR target positions (P1 to P4 or P1 to P3) with AP2/ERF *cis*‐regulatory elements. (e–h) ChIP‐qPCR data show an enrichment of chromatin DNA fragments at the indicated promoter region compared to NT plants. P1 to P4 in the *OsCML16* promoter (a, e), P1 to P3 in the *OsDREB1c* promoter (b, f) and *OsLEA24* promoter (c, g), and P1 to P3 in the *Os02g0771600* gene promoter as a negative control (d, h). The relative enrichment was normalized with total input. Values are the means** **± SD of three independent experiments. (i, j) Transient protoplast expression assay using a dual‐luciferase reporter system. (i) Schematic diagram of the reporter, internal control and two effector constructs. (j) Relative fLUC (fLUC/rLUC) activity in rice protoplasts. Values are the means** **±**** SD of three independent experiments.

We found that *OsCML16* was co‐expressed with 18 of the 56 candidate genes (Table 3), and that it was located in the centre of the co‐regulatory network (Figure 4a). We also identified many calmodulin‐binding motifs in the promoter regions of the genes in the *OsERF48* co‐regulatory network (Table 3). We therefore hypothesized that the drought‐induced OsERF48 binds directly to the *OsCML16* promoter, which then transduces a drought response to other downstream target genes. To examine the interaction between OsERF48 and the *OsCML16* promoter, we generated *myc*‐tagged *OsERF48* over‐expressing transgenic plants (*ROX‐Myc* ^*OsERF48*^) in order to conduct a chromatin immunoprecipitation (ChIP) assay (Figures S2c and S4). Three candidate genes, *OsCML16*,* OsLEA24/OsGolS2* and *OsDREB1c*, were selected for their high transcript levels (Figure 4b) and the presence of AP2/ERF *cis*‐regulatory elements in their promoter regions (Figure 5a–c). *Os02g0771600* was also chosen as a negative control, since it contains AP2/ERF *cis*‐regulatory elements in its promoter region (Figure 5d), yet it is absent from the network. The ChIP assay of the *ROX‐Myc* ^*OsERF48*^ roots revealed highly enriched genomic DNA fragments in all the *OsCML16* promoter positions (P1 to P4) containing AP2/ERF *cis*‐regulatory elements (Figure 5e). OsERF48 also bound to the P2 position of the *OsDREB1c* promoter with low affinity (Figure 5f). Genomic DNA fragments from the promoter regions of both *OsLEA24/OsGolS2* and *Os02g0771600* (negative control) were not enriched (Figure 5g, h).

To test whether the interaction of OsERF48 with the *OsCML16* promoter activates transcription of the latter, a transient protoplast expression assay using a dual‐luciferase reporter system was performed. *OsCML16p::fLUC* (firefly luciferase) and *CaMV35Sp::rLUC* (renilla luciferase) constructs were used as a reporter and an internal control, respectively. Two effector plasmids, *CaMV35Sp::OsERF48F* and *CaMV35Sp::OsERF48Δc1*, were transiently co‐expressed in rice protoplasts together with the reporter and the internal control, as indicated (Figure 5i, j). The OsERF48F (full‐length ORF) construct effectively activated the reporter gene expression, while the OsERF48Δc1 construct did not (Figure 5j), supporting our hypothesis that drought‐induced OsERF48 directly binds to the promoter of *OsCML16* via AP2/ERF *cis*‐acting regulatory elements, thereby activating its transcription.

## Discussion

*OsERF48* belongs to AP2/ERF group Ib, members of which contain four distinct conserved motifs (CMI‐1‐4). Here, we showed that the CMI‐1 motif is important for transcriptional activity using a yeast transactivation assay and a transient protoplast expression assay. The highly conserved acidic domain, EIDWD, found in the CMI‐1 region of OsERF48, appears to be essential for transactivation (Remacle *et al*., 1997) and is conserved in legume (*Medicago truncatula*), *A. thaliana* and maize (*Zea mays*) orthologs (Figure S8).

We showed that root‐specific overexpression of *OsERF48* is more effective than whole‐body overexpression in promoting root growth and enhancing drought tolerance. *ROX* ^*OsERF48*^ transgenic plants maintained their root growth, in terms of primary root length and lateral root density, under *in vitro* drought conditions (40% PEG‐infused media). Additionally, under normal growth conditions, the root dry weight of *ROX* ^*OsERF48*^ plants was twofold higher than that of NT plants. We propose that *OsERF48*‐mediated root modification enhances water uptake by increasing the total root surface area. Such robust root system‐mediated drought tolerance was previously observed in rice when *AtEDT1/HDG11* and *HYR* were overexpressed (Ambavaram *et al*., 2014; Yu *et al*., 2013). Deep rooting architecture caused by overexpressing *DEEP ROOTING 1* (*DRO1*), a QTL for deep rooting, in rice results in increased grain yield under drought conditions, by enhancing the capacity for water extraction from deep soil layers (Uga *et al*., 2013).

We found that, under field‐drought conditions, the *ROX* ^*OsERF48*^ plants produced a higher grain yield than the *OX* ^*OsERF48*^ and the NT control plants. Similar observations were made in our previous studies with whole‐body and root‐specific overexpression of *OsNAC*s (Jeong *et al*., 2010; Redillas *et al*., 2012) and *OsERF71* (Lee *et al*., 2016a) in rice. Root‐specific overexpressors always presented a higher grain yield than the whole‐body overexpressors and NT plants under field‐drought conditions. Moreover, those root‐specific overexpressors had similar levels of grain yield with NT plants under normal growth conditions. This was likely due to less of a trade‐off in the root‐specific overexpressors of the TFs than in the whole‐body overexpressors. In the current study, however, root‐specific overexpression of *OsERF48* showed defects in shoot growth and reduced grain yield under normal growth conditions. We believed that both whole‐body and root‐specific overexpression of *OsERF48* might have caused some negative effects on the normal growth of corresponding transgenic plants. Ito *et al*. (2006) also observed that the overexpression of *AP2/ERF* TFs showed shoot growth retardation of transgenic rice plants under normal growth conditions. A use of stress‐inducible promoter such as the *RD29A* promoter (Datta *et al*., 2012; Liu *et al*., 2014) could be an alternative way to avoid the growth defects.

We constructed a putative *OsERF48* regulatory network by cross‐referencing *ROX* ^*OsERF48*^ root RNA‐seq data with a co‐expression network database, from which we inferred the involvement of 20 drought‐related genes. These genes were associated with stress signalling, carbohydrate metabolism, cell‐wall proteins and drought response and included *OsCML16*, encoding a calmodulin‐like protein, which was located at the centre of our *OsERF48* co‐regulatory network, indicating an important regulatory role (Figure 4a). Many studies have suggested that CMLs are major calcium ion sensors and function in mediating plant stress tolerance (Magnan *et al*., 2008; Munir *et al*., 2016; Park *et al*., 2010; Xu *et al*., 2011). Other lines of evidence also indicate that downstream target genes regulated by CMLs include kinases, metabolic proteins, cytoskeletal proteins, ion channels and pumps, and TFs (Zeng *et al*., 2015). Indeed, similar categories of genes, such as TFs (*OsDREB1c*,* OsDERF5*,* OsAP2*‐39 and *AP37*), cell‐wall proteins (*OsXTH9*,* OsAGP24* and *OsAGP3*) and carbohydrate metabolic enzymes (*OsGolS1*,* OsGolS2*/*OsLEA24* and *RS5*), were included in the *OsERF48* co‐regulatory network. Most of these genes appear to be connected to *OsERF48* via *OsCML16*. These observations led us to propose that drought‐induced OsERF48 binds to the *OsCML16* promoter, which then interacts with other downstream target genes. This hypothesis was supported by the results of ChIP and transient protoplast expression assays, which indicated that OsERF48 binds to the promoter of, and activates, *OsCML16* (Figure 5e, j). Although *OsLEA24/OsGolS2* was up‐regulated in the *ROX* ^*OsERF48*^ plants, we found no evidence that it is a direct target of OsERF48, since we did not observe OsERF48 binding to its promoters (Figure 5g). We identified numerous calmodulin‐binding motifs in the promoter regions of the 20 genes in the co‐regulatory network (Table 3), suggesting possible interaction of OsCML16 with those promoters, and consequently involvement in the drought stress response.

We found evidence in the literature (Table 3) that other genes in the *OsERF48* co‐regulatory network are also closely associated with drought tolerance and root growth. For example, *OsDERF5*,* OsAP2‐39* and *AP37* were reported to be stress‐inducible *AP2/ERF* genes in rice (Oh *et al*., 2009), and *AP37* promotes crown root development, as well as its overexpression increases drought tolerance and grain yield under drought conditions (Oh *et al*., 2009; Zhao *et al*., 2015). Plant XTH proteins play a role in cell‐wall restructuring and in cell expansion during root growth (Vilches‐Barro and Maizel, 2015), and rice *XTH* and *XYLOSE ISOMERASE* genes were up‐regulated under water‐deficit conditions, where both were associated with maintenance of root growth in rice plants (Yang *et al*., 2006). RNA interference‐mediated suppression of *AtXTH18* in *A. thaliana* caused a reduction in root length and cell size (Osato *et al*., 2006), indicating its importance in root phenotype determination. Arabinogalactan proteins are plant cell‐wall glycoproteins that have been reported to be associated with various plant growth and developmental processes, such as cell expansion and proliferation, cell‐wall plasticization, salt tolerance and root growth (Seifert and Roberts, 2007). During early root development of cotton (*Gossypium hirsutum*), *GhAGP31* has been shown to be involved in the response to cold stress (Gong *et al*., 2012), and *OsXTH9* and *OsAGP3*, which we observed were both up‐regulated in *ROX* ^*OsERF48*^ roots and down‐regulated in *RNAi* ^*OsERF48*^ roots, have been shown to be involved in root growth and abiotic stress tolerance. Similarly, *OsGolS2/OsLEA24*,* OsGolS1* and *RS5* were also up‐ and down‐regulated in the *ROX* ^*OsERF48*^ and the *RNAi* ^*OsERF48*^ roots, respectively (Figure 4a, b). *GolS* and *RS* are key enzymes in the biosynthesis of raffinose family oligosaccharides (RFO), such as raffinose, stachyose and galactinol. Overexpression plants of the genes encoding the enzymes involved in RFO biosynthesis, such as *GolS* and *RS* were shown to result in intracellular accumulation of RFO, which serve as osmoprotectants and may aid in drought tolerance (Shimosaka and Ozawa, 2015; Taji *et al*., 2002; Zhuo *et al*., 2013). Overexpression of *OsWRKY11* was shown to cause an up‐regulation of *OsGolS1*,* OsLEA24/OsGolS2* and *RS5*, resulting in elevated raffinose levels and increased drought tolerance (Wu *et al*., 2009). These reports suggest that up‐regulation of the *OsGolS1*,* OsLEA24/OsGolS2* and *RS5* in *ROX* ^*OsERF48*^ plants may trigger the accumulation of RFO in roots, which is consistent with the *ROX* ^*OsERF48*^ plants having a drought tolerance.

Endogenous levels of ABA and Ca^2+^ increase upon exposure to various abiotic stresses. Many genes regulated by ABA are therefore involved in the Ca^2+^ signal transduction (Tuteja, 2007). Conversely, genes that are not responsive to ABA are also involved in Ca^2+^ signalling. For example, overexpression of *OsCML4*, a calcium‐signalling gene that is not responsive to ABA, conferred drought tolerance via ROS‐scavenging (Yin *et al*., 2015). Similarly, our ABA‐independent *OsERF48* regulated the *OsCML16* which resulted in drought tolerance through enhanced root growth. Together, these observations support that the ABA‐dependent and ABA‐independent pathways cross‐talk and Ca^2+^ is a common second messenger of the crosstalk during abiotic stresses (Roychoudhury *et al*., 2013).

We have demonstrated that *OsERF48* overexpression enhances root growth and drought tolerance under drought conditions. We constructed a putative co‐regulatory network of *OsERF48* by cross‐referencing *ROX* ^*OsERF48*^ root RNA‐seq data with a co‐expression network database. A number of genes that are known to confer drought tolerance were present in the network. The calmodulin‐like protein gene, *OsCML16*, a direct target of OsERF48, appears to transduce OsERF48 actions to downstream target genes that together confer the acquired root phenotype and drought tolerance of the *ROX* ^*OsERF48*^ plants. In addition, we present evidence of a role for OsERF48 as an upstream regulator of *OsCML16*.

## Experimental procedures

### Plasmid construction for rice transformation

To generate *OsERF48* overexpressing transgenic rice, the *OsERF48* (Os08g0804500) coding sequence was amplified from rice (*O. sativa cv* Ilmi) cDNA using a high‐fidelity DNA polymerase PrimeSTAR (TaKaRa, Kyoto, Japan). The amplified *OsERF48* fragment was ligated into the *pPZP‐PGD1* vector for whole‐body expression (*OX* ^*OsERF48*^) (Park *et al*., 2012) and *pPZP‐RCc3* for root‐specific expression (*ROX* ^*OsERF48*^) (Jeong *et al*., 2010). For the RNAi (*RNAi* ^*OsERF48*^) construct, a 289 bp *OsERF48* fragment (−137 to +152 from the translational start ATG) was sub‐cloned into the *pENTRd‐TOPO* vector (Invitrogen, Carlsbad, CA) and transferred to the *pGOS2‐RNAi* vector (Lee *et al*., 2016a) using the Gateway cloning system (Invitrogen). For the *myc*‐tagged *OsERF48* (*ROX‐Myc* ^*OsERF48*^) construct, the *OsERF48* coding sequence was fused to 3′ end of the sequence encoding a *6xMyc* tag in the *pE3n* vector (Dubin *et al*., 2008), and the fused fragment was transferred to the *p700RCc3* vector (Jeong *et al*., 2010). Transgenic rice (*O. sativa cv* Ilmi) was obtained by *Agrobacterium tumefaciens* (EHA101 for *ROX* ^*OsERF48*^ and *OX* ^*OsERF48*^; LBA4404 for *RNAi* ^*OsERF48*^ and *ROX‐Myc* ^*OsERF48*^)‐mediated transformation. To verify the copy number, a Southern blot was performed as previously described (Lee *et al*., 2016b). Vector maps and primer sequences used in this study are listed in Figure S1 and Table S3, respectively.

### Abiotic stress treatment and qRT‐PCR analysis

Rice (*O. sativa* cv. Ilmi) seeds were germinated on Murashige‐Skoog (MS) medium (Duchefa Biochemie, Haarlem, Netherlands), transferred to soil and grown for 14 days in a green house at 28 °C. For abiotic stress treatments, soil was removed from the roots of the seedlings, and drought stress was induced by air‐drying the seedlings, while salinity stress and ABA treatment were imposed by incubating the seedlings in water containing 400 mm NaCl and 100 μm ABA, respectively at 28 °C. Low temperature stress was induced by incubating seedlings in water and placing them inside a cooler at 4 °C. Leaves and roots from the stress‐treated plants were sampled at the indicated time points. Total RNA was extracted from rice leaves or roots using the TRIzol reagent (Invitrogen) in accordance with the manufacturer's instructions.

cDNA was synthesized using Revertaid™ reverse transcriptase (ThermoFisher Scientific, Waltham, MA), and real‐time PCR analysis was performed using the Solg™ 2× real‐time PCR smart mix with evagreen (Solgent, Seoul, Korea) with a Mx3000P real‐time PCR system (Agilent Technologies, Palo Alto, CA). The *OsUbi1* gene (Os06g0681400) was used as an internal standard, and three biological replicates were analysed. Values are the means ± SD (standard deviation) of three independent experiments. All primer sequences are listed in Table S3.

### Drought tolerance evaluation at the vegetative stage

*OsERF48* transgenic and NT control plants (*O. sativa* cv. Ilmi) were germinated on MS media at 28 °C for 3 days. Thirty plants from each line were transplanted into ten soil pots (4 × 4 × 6 cm) within a container (59 × 38.5 × 15 cm; three plants per pot) and grown for 4 weeks in a greenhouse at 28–30 °C. Pots were moved from the container for a 4‐day drought treatment and returned into the container for re‐watering until the plants recovered.

To measure chlorophyll fluorescence and the performance index, 2‐week‐old plants were transplanted into a 15‐cm‐diameter × 14‐cm‐tall pot within another larger container (66 × 45.3 × 22.5 cm) and grown for 2 months. By removing the pots from the container, drought stress was performed for 6 days. After a 1‐h dark adaptation, the longest leaves from each plant were selected and measured at their apex, middle and base regions using the Handy‐pea fluorimeter (Hansatech Instrument, Norfolk, UK). Thirty readings per line were averaged using the Handy‐pea software (version 1.31). Chlorophyll *a* fluorescence (*F*_v_/*F*_m_) and the performance index were measured and analysed according to the equations of the JIP test (Redillas *et al*., 2011). Drought‐induced symptoms were visualized using a NEX‐5N camera (Sony, Kyoto, Japan), and soil moisture was measured using a SM150 soil moisture sensor (Delta T Devices, Cambridge, UK) at the indicated time points.

### Field‐drought tolerance evaluation at the reproductive stage

Evaluation of yield components of transgenic and nontransgenic (NT) plants was performed in the rice paddy field at Kyungpook National University, Gunwi (128:34E/36:15N), Korea. A randomized design was introduced for three replicates using three different 10‐m^2^ plots. Thirty seedlings were planted in the paddy field for normal growth, and 18 seedlings were planted in a pot (15 cm diameter × 14 cm tall) for field‐drought conditions. All plants were applied with fertilizer at 70N/40P/70K kg/ha after the last paddling. To make the field‐drought conditions, plants were grown in a tank with a rain‐off shelter to cover rice plants from rain. During 10 days before and after heading, intermittent drought stress was applied by draining water from the tank. When complete leaf‐rolling was observed in the plants after the first drought treatment, they were irrigated overnight and allowed to recover. Plants were then subjected to the second round of drought treatment until another complete leaf‐rolling occurred. After two times of drought stress treatments, all plants were irrigated until harvest. Yield parameters were scored with the 30 and 18 plants per each line from three different plots for normal and drought field conditions, respectively. The results were compared with those of NT controls, using analysis by SPSS software.

### Characterization of root phenotypes

Seeds were immersed in water at 37 °C for 1 day, sowed on 1/2 MS medium containing 1% agar without sucrose and grown vertically for 1 day in the dark and 5 days under a 16 : 8 light : dark photoperiod at 28 °C. For *in vitro* drought conditions, polyethylene glycol (PEG)‐infused plates were prepared by dissolving solid PEG8000 in a sterilized solution of 1/2 MS medium with 6 mm 4‐morpholineethanesulfonic acid hydrate (MES) buffer (pH 5.7), followed by overlaying of the PEG solution onto 1.5% agar‐solidified half‐strength MS medium with 6 mm MES buffer (pH 5.7). The agar medium and PEG solution were equilibrated for at least 12 h, and the excess PEG solution was removed before use. Drought stress strength was considered to be proportional to the concentration of the overlaid PEG solution: 0% (control); 25% and 40% (drought stress; Verslues *et al*., 2006). To measure primary root length and lateral root number, images were taken using a NEX‐5N camera (Sony, Kyoto, Japan) and analysed with ImageJ software (https://imagej.nih.gov). The number of lateral roots was counted within a 3‐ to 4.5‐cm region from the root tip using a stereomicroscope (Leica, Bensheim, Germany).

### Protein subcellular localization using rice protoplasts

The *OsERF48*,* OsNF*‐YA7, *GFP* and *mCherry* coding regions without stop codons were amplified using the high‐fidelity DNA polymerase PrimeSTAR (TaKaRa) and specific primers (Table S3). Using the In‐fusion cloning system (TaKaRa), multiple PCR products (*OsERF48* and *GFP* for the *OsERF48*‐*GFP* construct and *OsNF‐YA7* and *mCherry* for the *OsNF‐YA7‐mCherry* construct) were cloned into the *pHBT* vector (GenBank accession number EF090408), which harboured the constitutive 35S promoter. These vectors were transiently expressed in rice protoplasts, as previously described (Jung *et al*., 2015). Eighteen hours after transformation, GFP and mCherry fluorescence were observed using a SP8 STED laser scanning confocal microscope (Leica, Bensheim, Germany).

### Transactivation assay in yeast

Five OsERF48 mutants with conserved motifs (CMI‐1‐4) separately deleted (Figure S2) were produced using the high‐fidelity DNA polymerase PrimeSTAR (TaKaRa). Primer combinations and sequences are listed in Table S3. Each deletion fragment was cloned into the *pGBKT7* vector (Clontech, Palo Alto, CA) using the In‐fusion cloning system (TaKaRa). All of the mutant vectors were transformed into the Y2HGold yeast strain through LiAc‐mediated transformation according to the manufacturer's instructions (Clontech). Transformants were cultured at 30 °C on Synthetic Dropout (SD)/‐Trp and SD/Trp with 200 ng/mL of aureobasidin A (AbA) and 40 μg/mL of X‐α‐gal.

### RNA‐seq analysis

Total RNA was extracted from 2‐week‐old transgenic and nontransgenic (NT) roots using a RNeasy plant kit and treated with DNase I according to the manufacturer's instructions (Qiagen, Hilden, German). RNA samples from two biological replicates with two technical replicates each were prepared, from the two different transgenic lines (*ROX#2* and *ROX#6*). Processing of library construction, next‐generation sequencing (NGS) sequencing and DEG analysis was performed by Macrogen Inc., Seoul, Korea. DEGs were defined by an expression change ≥2‐fold with a *P* value <0.05. For validation of candidate genes from the RNA‐seq analysis, RT‐PCR was performed of root material from 2‐week old transgenic and WT plants, prepared separately from the RNA‐seq sample. All primer sequences are listed in Table S3.

### ChIP‐qPCR assay

Two‐week‐old *ROX‐Myc* ^*OsERF48*^ and NT plants grown on soil were hydroponically adapted in water for 3 days and then fixed by cross‐linking with 1% formaldehyde under vacuum for 15 min. Cross‐linking was stopped by the addition of glycine to a final concentration of 125 mm and application of vacuum for 10 min. After washing the plants in cold water, roots were collected and frozen in liquid nitrogen, and stored at −80 °C. The ChIP assay was performed as described by Chung *et al*. (2009), except that an anti‐myc antibody (SC‐789; Santa Cruz Biotech, Santa Cruz, CA) used. The ChIP product was analysed via quantitative PCR on a Mx3000P real‐time PCR system (Agilent Technologies). The enrichment values were normalized to the input sample. Values are the means ± SD of three independent experiments. All primer sequences are listed in Table S3.

### Protoplast isolation and transactivation assay

The *OsERF48F*,* OsERF48Δc1* and a promoter region of *OsCML16* were amplified by PCR using a high‐fidelity DNA polymerase PrimeSTAR (TaKaRa). For effector constructs, *OsERF48F* and *OsERF48Δc1* were cloned into the *pHBT* vector (GenBank accession number EF090408) containing the 35S promoter, and for the reporter construct, the *OsCML16* promoter region was cloned into the *pGST6‐LUC‐NOS* vector (GenBank accession number EF090412.1) using the In‐fusion cloning system (TaKaRa). Protoplast isolation from shoots of 10‐day‐old rice seedlings (*O. sativa* cv. Ilmi) and PEG‐mediated transformation were performed as previously described (Jung *et al*., 2015). Fifteen microlitres of vector solution, including 3 μg of effector, 1 μg of reporter and 1 μg of internal control were transfected into the isolated protoplast solution harbouring up to 3.5 × 10^6^ cells. Dual‐luciferase activity was analysed using the dual‐luciferase reporter assay system (Promega, Madison, WI) and measured with an Infinite M200 system (Tecan Systems, San Jose, CA). Three independent transfections for each sample were performed, and the relative luciferase activity was calculated as the ratio between fLUC and rLUC. The *35S::rLUC* construct was used as an internal control. Primer sequences are listed in Table S3.

## Conflict of interest

The authors declare no conflict of interest.

## Supporting information


**Figure S1** Predicted domain and motifs of OsERF48 and their nucleotide and amino acid sequences.
**Figure S2** Vectors used for rice transformation.
**Figure S3** Southern blot analysis of *OsERF48* overexpression lines.
**Figure S4** Drought tolerance of transgenic and nontransgenic (NT).
**Figure S5** Vigorous root growth in *OsERF48* overexpressors.
**Figure S6** Transcriptome profile of *ROX* ^*OsERF48*^ roots compared with nontransgenic (NT) roots.
**Figure S7** Co‐expression matrix of 56 candidate genes identified amongst the differentially expressed genes (DEGs) in *ROX* ^*OsERF48*^ roots compared to wild type using the RiceFREND web tool (http://ricefrend.dna.affrc.go.jp/). Red boxes indicate pairings of each gene.
**Figure S8** Alignment of amino acid sequences from the CMI‐1 region of OsERF48 and orthologs.
**Table S1** Fifty‐six candidate genes identified from the differentially expressed genes (DEGs) from the RNA‐seq analysis of *ROX* ^*OsERF48*^ roots.
**Table S2** Nodes (genes) constituting the *OsERF48* co‐regulatory network.
**Table S3** Primers used in this study.
