# Supplementary material for: Overexpression of OsERF48 causes regulation of OsCML16, a calmodulin‐like protein gene that enhances root growth and drought tolerance
Source: Plant Biotechnol J. 2017 Mar 27;15(10):1295–308. doi: 10.1111/pbi.12716 (PMC5595718; doi:10.1111/pbi.12716)
Supplement: Supplementary file 1 — Figure S1 Predicted domain and motifs of OsERF48 and their nucleotide and amino acid sequences. Figure S2 Vectors used for rice transformation. Figure S3 Southern blot analysis of OsERF48 overexpression lines. Figure S4 Drought tolerance of transgenic and nontransgenic (NT). Figure S5 Vigorous root growth in OsERF48 overexpressors. Figure S6 Transcriptome profile of ROX OsERF48 roots compared with nontransgenic (NT) roots. Figure S7 Co‐expression matrix of 56 candidate genes identified amongst the differentially expressed genes (DEGs) in ROX OsERF48 roots compared to wild type using the RiceFREND web tool (http://ricefrend.dna.affrc.go.jp/). Red boxes indicate pairings of each gene. Figure S8 Alignment of amino acid sequences from the CMI‐1 region of OsERF48 and orthologs. Table S1 Fifty‐six candidate genes identified from the differentially expressed genes (DEGs) from the RNA‐seq analysis of ROX OsERF48 roots. Table S2 Nodes (genes) constituting the OsERF48 co‐regulatory network. Table S3 Primers used in this study. [file PBI-15-1295-s001.docx]

**Overexpression of *OsERF48* causes regulation of *OsCML16*, a calmodulin-like protein gene that enhances root growth and drought tolerance**

Harin Jung^1,2^, Pil Joong Chung^1^, Su-Hyun Park^1,3^, Mark Christian Felipe Reveche Redillas^1^, Youn Shic Kim^1^, Joo-Won Suh^2,^ *, and Ju-Kon Kim^1,^ *

^1^Graduate School of International Agricultural Technology and Crop Biotechnology Institute/GreenBio Science and Technology, Seoul National University, Pyeongchang 25354, Korea.

^2^Center for Nutraceutical and Pharmaceutical Materials, Myongji University, Yongin, Gyeonggi, 17058, Korea.

^3^Present address: Laboratory of Plant Molecular Biology, Rockefeller University, New York, New York 10065, USA.

***Correspondence:** Ju-Kon Kim* and Joo-Won Suh*


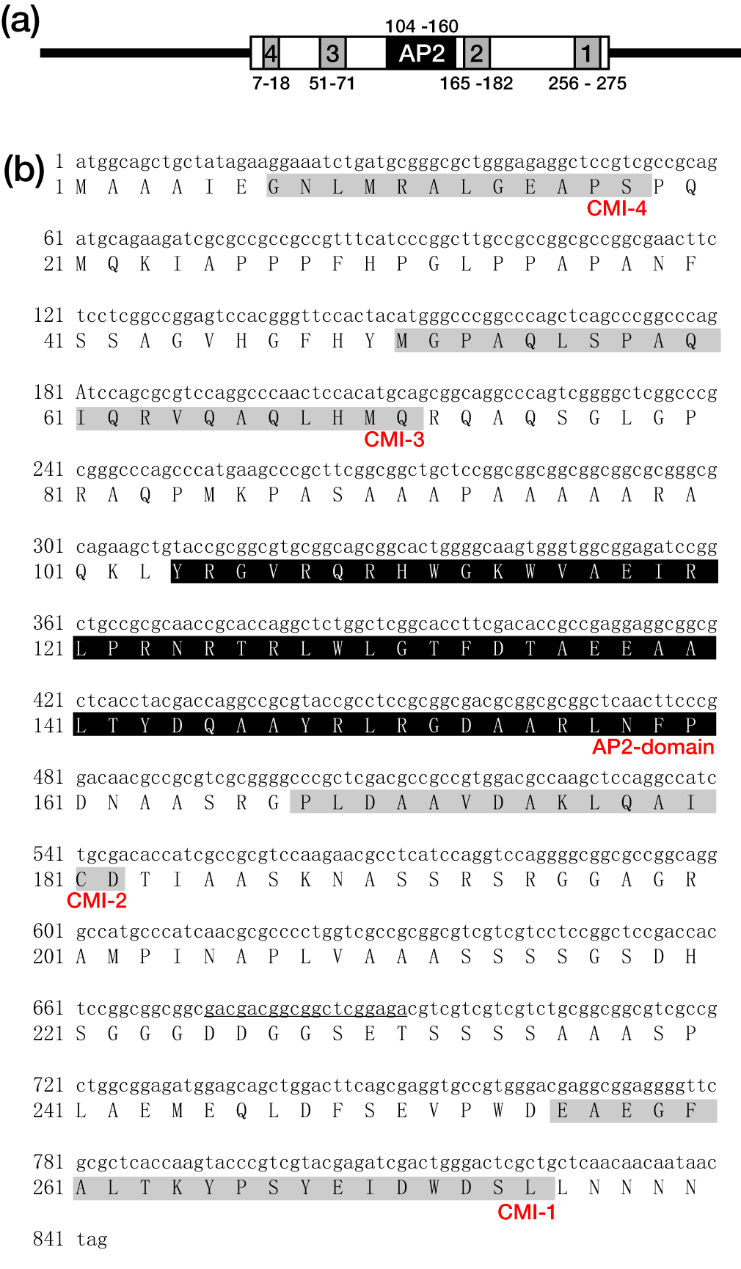


Figure S1. Predicted domain and motifs of OsERF48 and their nucleotide and amino acid sequences.

(a) Predicted domains and motifs of OsERF48, based on Nakano *et al*. (2006). AP2 indicates an AP2/ERF domain. Grey boxes with numbers are the four conserved motifs in group I AP2/ERF family proteins (CMI-1,2,3 and 4); numbers above or below each domain indicate the amino acid positions of these domains and motifs; Black line, 5’ and 3’ untranslated region. (b) Nucleotide and amino acid sequence of OsERF48. Small letter, nucleotide sequence; capital letter, amino acid sequence; black box, AP2/ERF domain; grey box, conserved motifs (CMI-1,2,3 and 4).


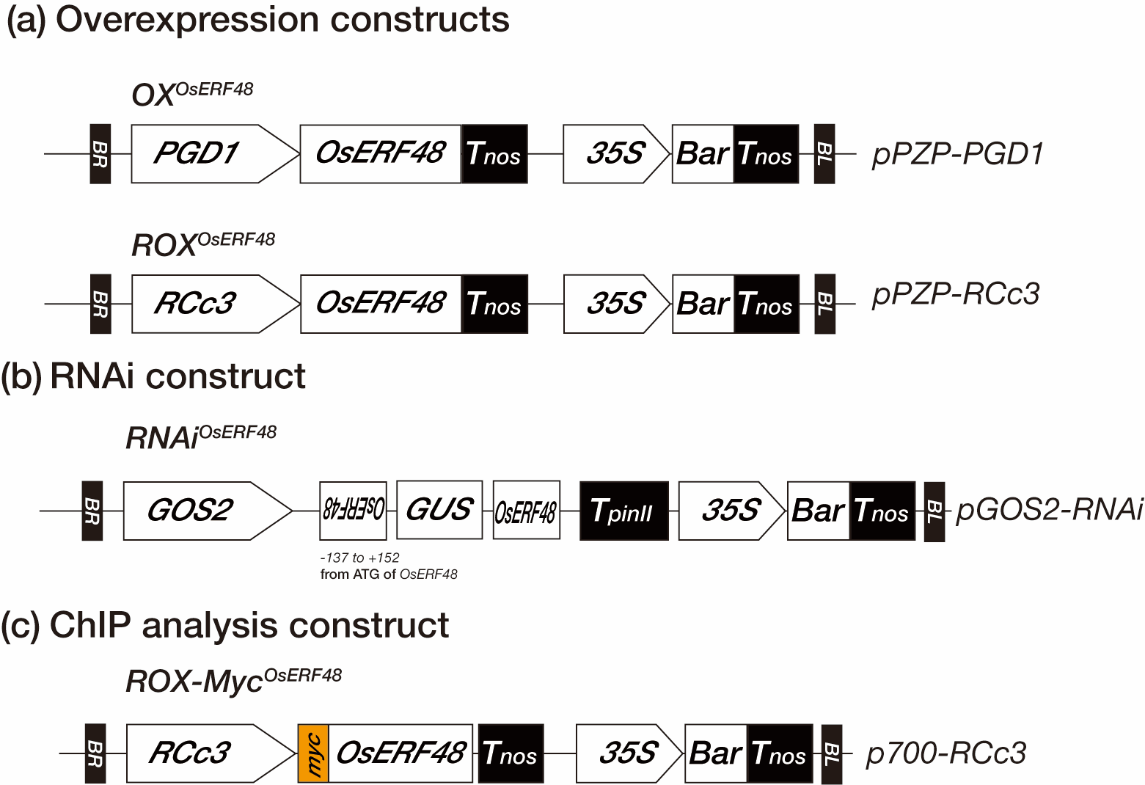


Figure S2. Vectors used for rice transformation.

(a) For *OsERF48*: whole-body (*OX^OsERF48^*) and root-specific overexpression (*ROX^OsERF48^*). (b) For the RNA interference construct, *RNAi^OsERF48^*. (c) For the myc-tagged version of *OsERF48*- *ROX-Myc^OsERF48^* *OsERF48, OsERF48* coding region; *PGD1,* promoter of rice *Cytosolic 6-phosphogluconate dehydrogenase* (Os06g0111500); *RCc3*, promoter of rice *lipid transfer protein-like* (Os02g0662000); *Tnos,* the 3’ region of *nopaline synthase* gene; *TpinII*, 3’ region of the potato (*Solanum tuberosum*) *proteinase inhibitor II* gene; *Bar*, a *herbicide-resistant selection marker*; *35S*, 35S promoter of *Cauliflower mosaic virus*; BL, left border; BR, right border.


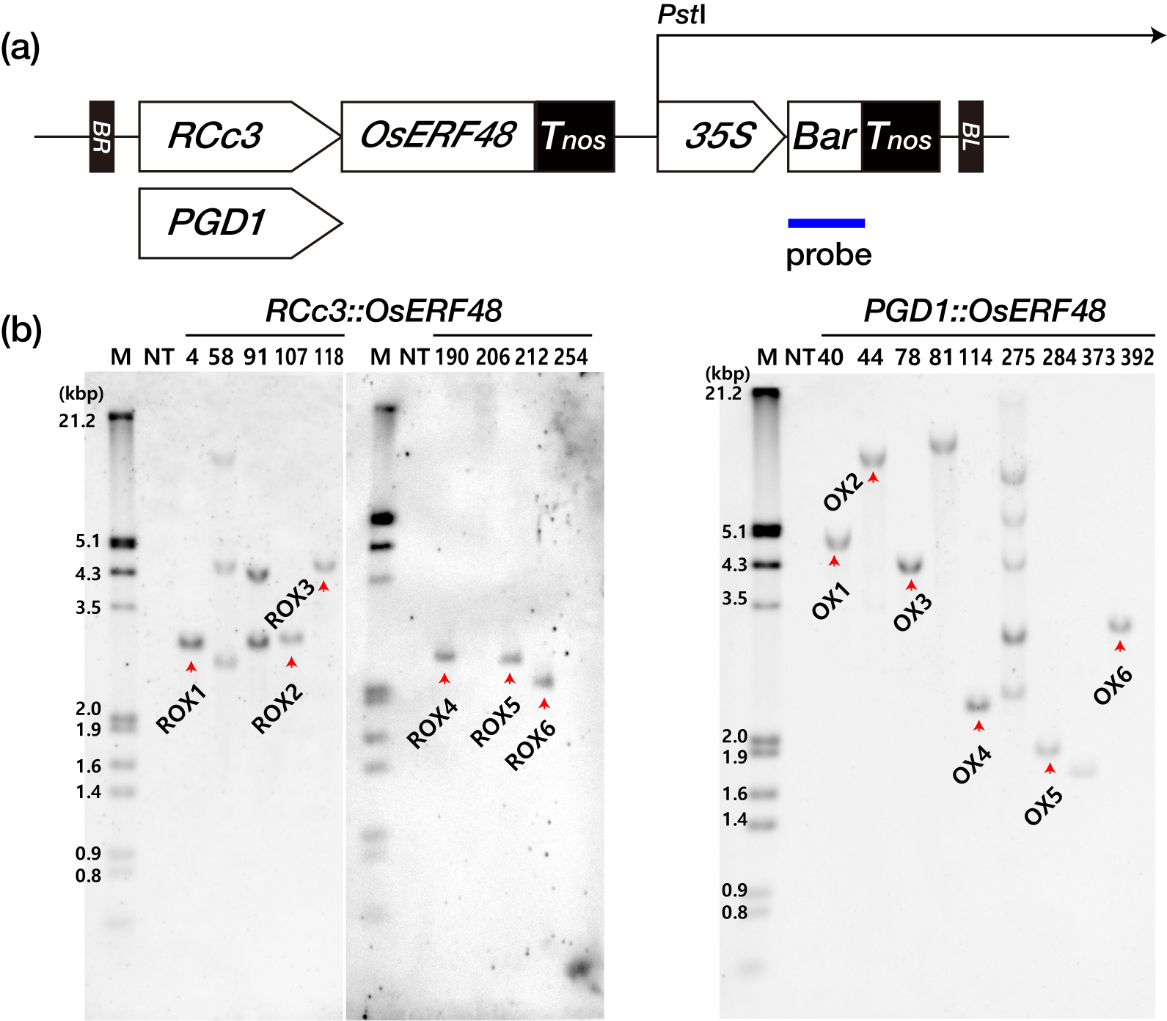


Figure S3. Southern blot analysis of *OsERF48* overexpression lines.

Five micrograms of genomic DNA from T_3_ transgenic and nontransgenic (NT) control plants was used after digestion with *Pst*I. (a) Single DIG-labeled probes corresponding to the *35S* promoter and *Bar* gene were designed for two overexpression vectors. (b) Southern blot analysis with the *Bar* probe. M, DNA marker. Red arrows indicate single copy band.


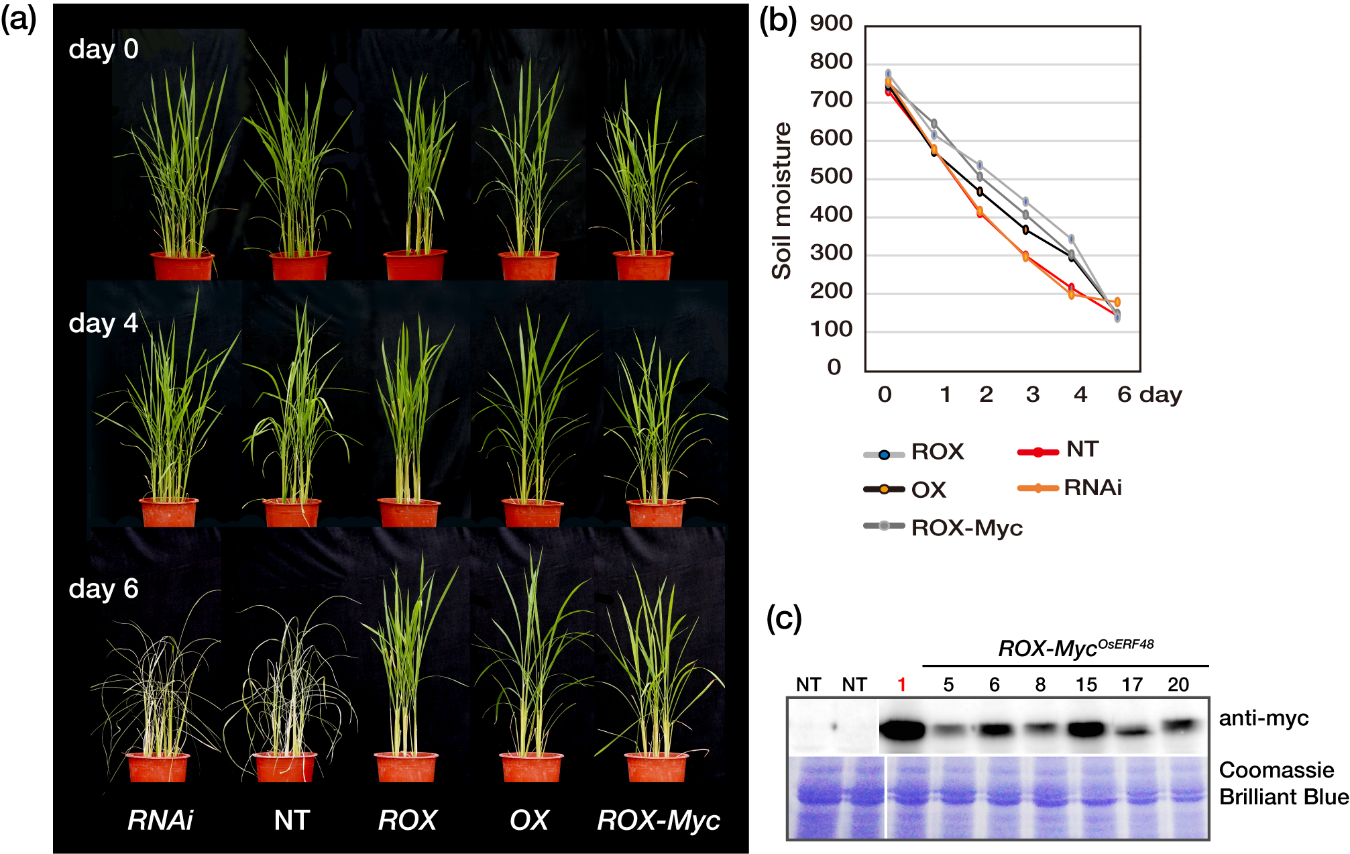


Figure S4. Drought tolerance of transgenic and nontransgenic (NT).

(a) Drought-tolerance of *OsERF48* transgenic and NT plants. All plants were grown in soil for 2 months under well-watered conditions and exposed to drought stress for 6 days. (b) Soil moisture of the pots exposed to drought treatment at the indicated time points. Values are the means ± SD (n=20). (c) Myc-OsERF48 protein expression in *ROX-Myc^OsERF48^* transgenic plants shown by Western blot analysis with an anti-myc antibody. NT indicates nontransgenic control plants. The upper panel shows Myc-OsERF48 recombinant proteins in the blot and the lower panel shows Coomassie Brilliant Blue staining as a loading control for each line.


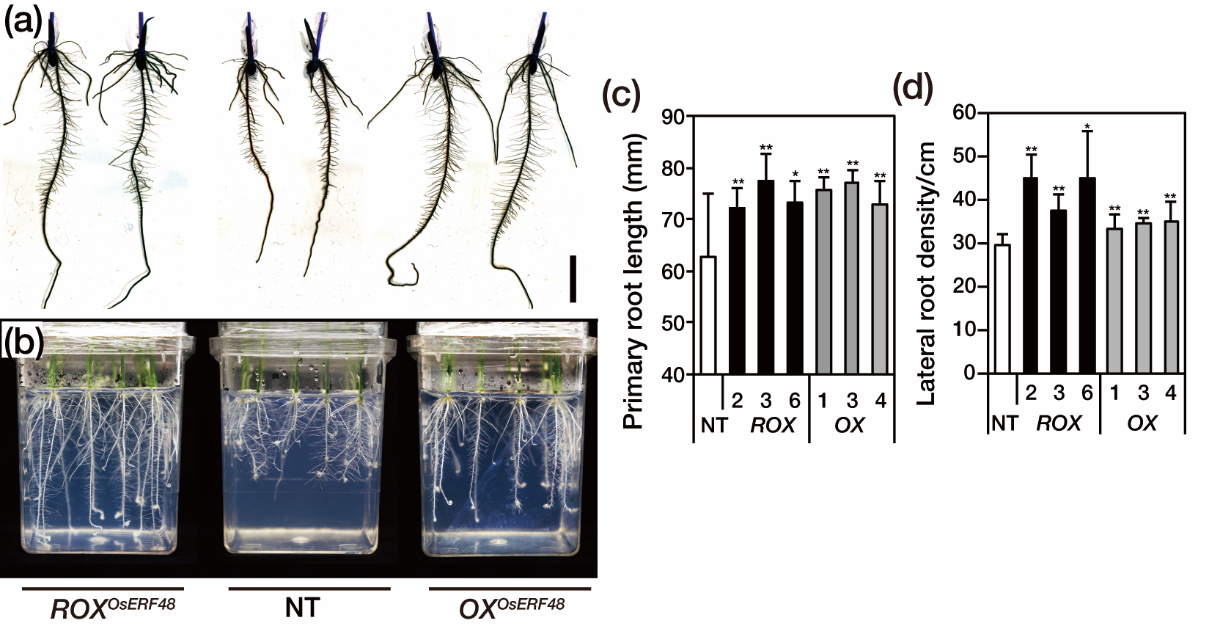


Figure S5. Vigorous root growth in *OsERF48* overexpressors.

Transgenic and nontransgenic (NT) plants were grown vertically in a square plates and growth boxes containing 1/2 MS media for 5 days. (a) The transgenic plants had longer and more branched root growth than the NT plants when grown in square plates. (b) Deeper root growth of transgenic plants compared to NT plants when grown in a growth box. (c) Length of primary roots of transgenic and NT plants grown in square plates. (d) Lateral root density in primary roots of transgenic and NT plants grown in square plates. Each data point represents the mean ± SD with biological replicates (n = 12) of 3 independent transgenic and NT control plants. *ROX*, *ROX^OsERF48^*; *OX*, *OX^OsERF48^.*

**
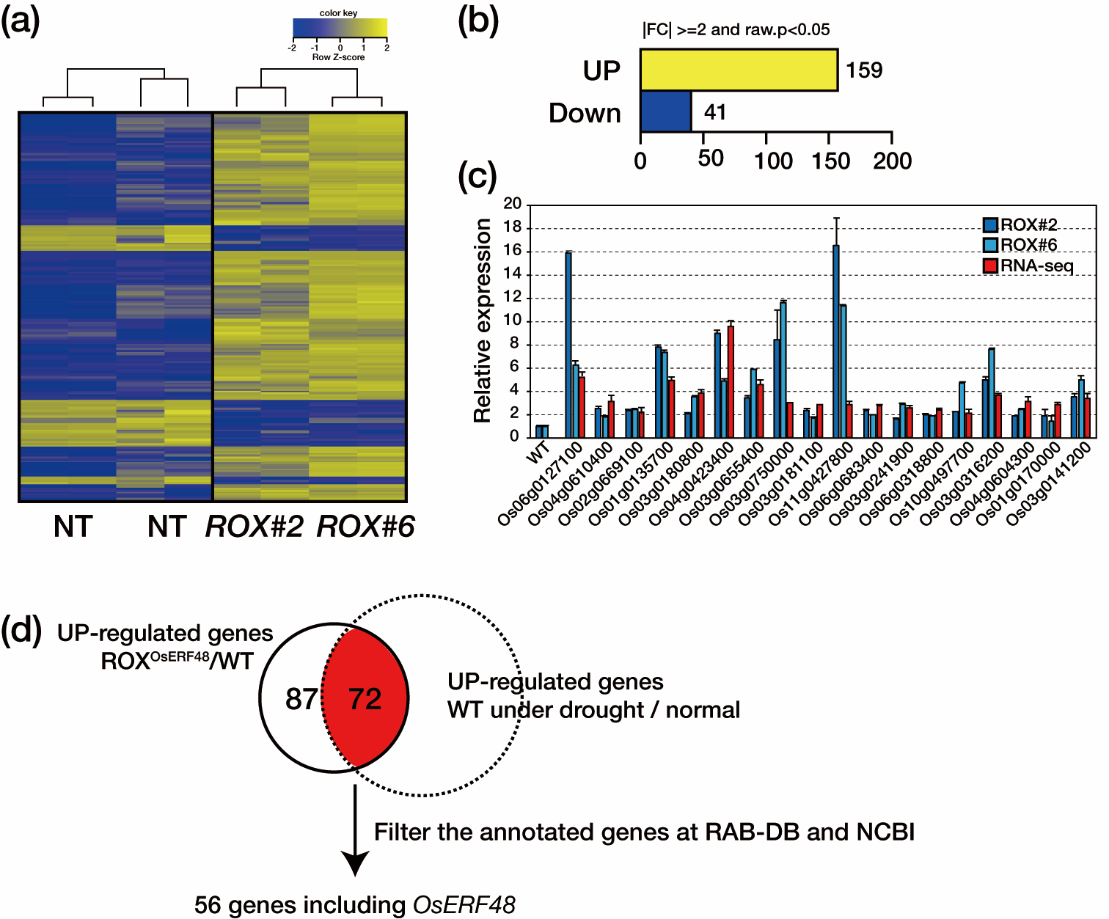
**

Figure S6. Transcriptome profile of *ROX^OsERF48^* roots compared with nontransgenic (NT) roots.

(a) Hierarchical clustering analysis of all differentially expressed genes (DEGs) based on expression levels. (b) Up- and down-regulated genes amongst the DEGs identified in *ROX^OsERF48^* roots. (c) qRT-PCR validation of 18 up-regulated genes using roots of 2-week-old *ROX^OsERF48^* lines (*ROX#2* and *ROX#6*). (d) Venn diagram of up-regulated genes amongst the DEGs identified in *ROX^OsERF48^* roots and DEGs identified in drought-treated NT roots from public data (TENOR: http://tenor.dna.affrc.go.jp/). *ROX*, *ROX^OsERF48^.*


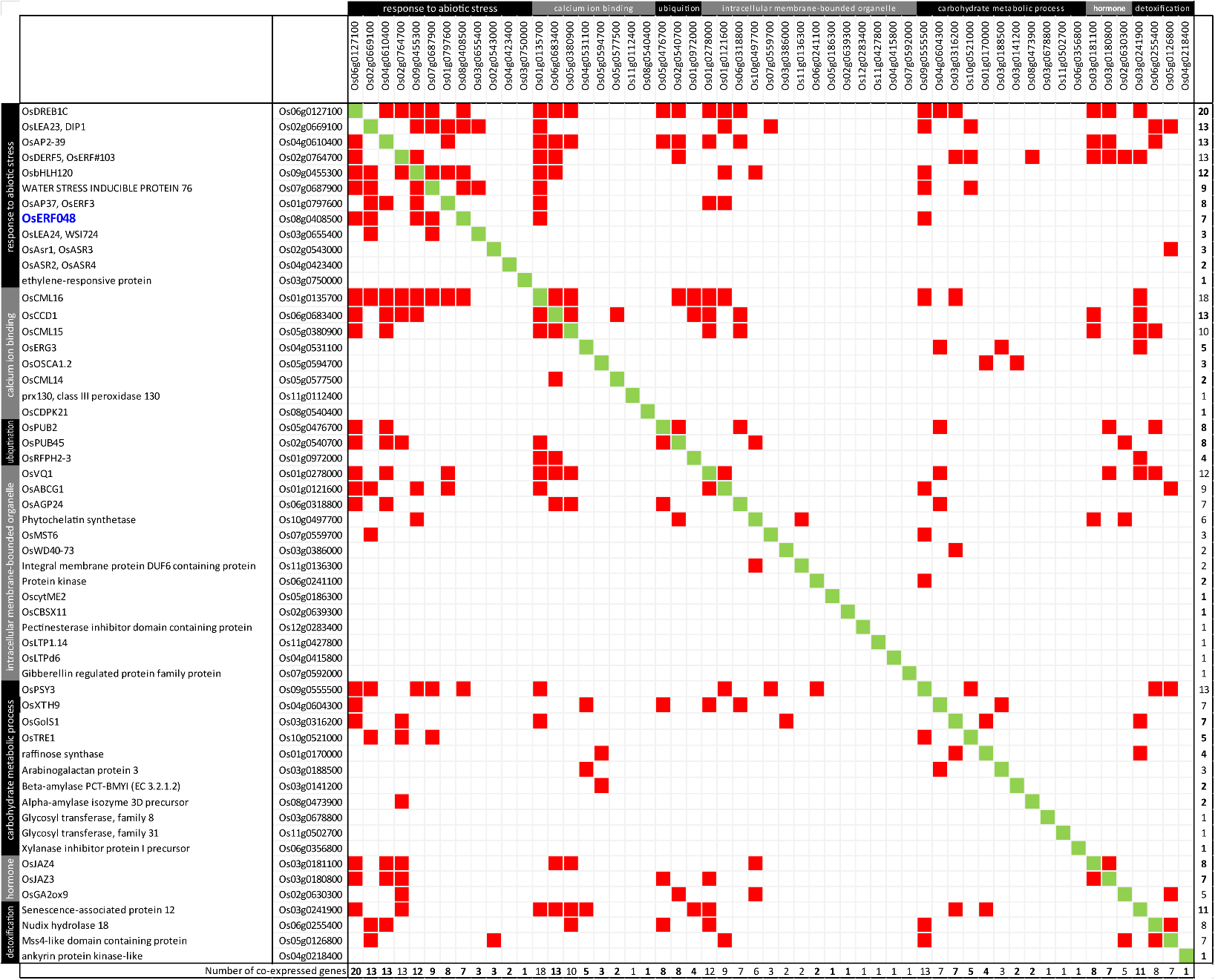


Figure S7. Co-expression matrix of 56 candidate genes identified amongst the differentially expressed genes (DEGs) in *ROX^OsERF48^* roots compared to wild type using the RiceFREND web tool (http://ricefrend.dna.affrc.go.jp/). Red boxes indicate pairings of each gene.

**
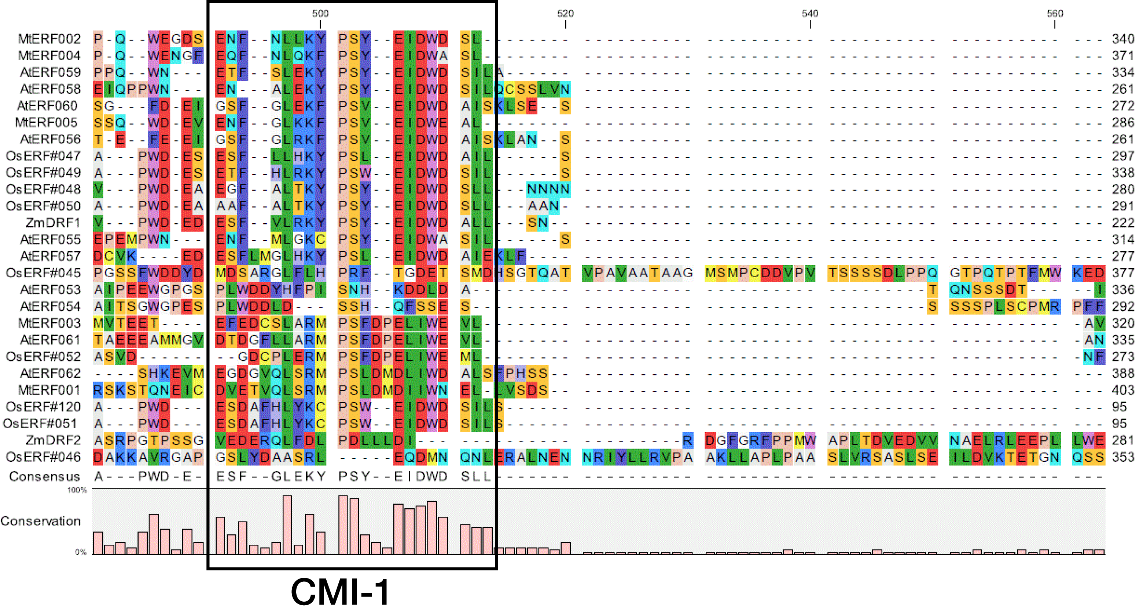
**

Figure S8. Alignment of amino acid sequences from the CMI-1 region of OsERF48 and orthologs.

OsERF48 orthologs from various plant species. We included group I from *Arabidopsis* *thaliana*, rice (*Oryza sativa*), legume (*Medicago truncatula*) and maize (*Zea mays*); 5 legume (MtERF0001; MtERF0002 (WXP2); MtERF0003; MtERF0004 (WXP1) and MtERF0005)*,*10 *A. thaliana* (AtERF053; AtERF054; AtERF055; AtERF056; AtERF057; AtERF058; AtERF059; AtERF060; AtERF061 and AtERF062), 9 rice (OsERF045, OsERF046, OsERF047, OsERF048, OsERF049, OsERF050, OsERF120, OsERF151and OsERF052) and 2 maize (DRF1 and DRF2) protein sequences were used for the multiple sequence alignment performed with the CLC sequence viewer software (https://www.qiagenbioinformatics.com). The black box indicates the CM1-1 region of OsERF48.

Table S1. Fifty-six candidate genes identified from the differentially expressed genes (DEGs) from the RNA-seq analysis of *ROX^OsERF48^* roots.

|  | Description | ID | ROX/WT | ROX/WT | Dro/C_3h |
| --- | --- | --- | --- | --- | --- |
|  |  |  | fc. | pval | fc. |
| Transcription Factors | | | | | |
|  | ^2^OsASR2, OsASR4 | Os04g0423400 | 9.6 | 0.00 | 3.8 |
|  | ^1,2^OsDREB1C | Os06g0127100 | 5.2 | 0.01 | 5.2 |
|  | ^1^OsDERF5, OsERF103 | Os02g0764700 | 4.2 | 0.01 | 4.6 |
|  | ^1,2^OsAP2-39 | Os04g0610400 | 3.1 | 0.03 | 4.0 |
|  | OsbHLH120 | Os09g0455300 | 2.8 | 0.01 | 5.7 |
|  | OsAsr1, OsASR3 | Os02g0543000 | 2.3 | 0.00 | 1.1 |
|  | ^1^OsAP37, OsERF3 | Os01g0797600 | 2.2 | 0.02 | 2.9 |
| Signal transduction | | | | | |
|  | ^1,2^calmodulin-like protein 16 (OsCML16) | Os01g0135700 | 4.9 | 0.00 | 6.0 |
|  | calmodulin-like protein 15 (OsCML15) | Os05g0380900 | 3.0 | 0.02 | 3.2 |
|  | ^1,2^EF-hand Ca2+-binding protein CCD1. (OsCCD1) | Os06g0683400 | 2.8 | 0.04 | 3.7 |
|  | C2 calcium-dependent membrane targeting domain containing protein (OsERG3) | Os04g0531100 | 2.4 | 0.00 | 1.7 |
|  | OsRFPH2-3, RING finger protein OsRFPH2-3 | Os01g0972000 | 2.4 | 0.02 | 2.3 |
|  | plant U-box-containing protein 2 (OsPUB2) | Os05g0476700 | 2.2 | 0.03 | 3.0 |
|  | Calcium-dependent protein kinase. (OsCDPK21) | Os08g0540400 | 2.2 | 0.00 | 2.8 |
|  | plant U-box-containing protein 45 (OsPUB45) | Os02g0540700 | 2.1 | 0.01 | 3.6 |
|  | Hyperosmolality-gated calcium-permeable channel 1.2 (OsOSCA1.2) | Os05g0594700 | 2.0 | 0.01 | 1.3 |
|  | calmodulin-like protein 14 (OsCML14) | Os05g0577500 | 2.0 | 0.01 | 2.0 |
| Osmoprotectant/ response to stimulus | | | | | |
|  | WSI724 | Os03g0655400 | 4.5 | 0.00 | 2.3 |
|  | ^1^OsLEA23, Dehydration-stress inducible protein 1 | Os02g0669100 | 2.2 | 0.00 | 2.4 |
|  | OsTRE, OsTRE1 | Os10g0521000 | 2.1 | 0.00 | 3.2 |
|  | universal stress protein A-like protein | Os03g0750000 | 3.0 | 0.00 | 3.1 |
|  | Salt responsive WD40 protein5 (SRWD5, OsWD40-73) | Os03g0386000 | 2.4 | 0.00 | 2.4 |
| Intracellular membrane-bounded organelle (Cell wall remodeling) | | | | | |
|  | Protein kinase | Os06g0241100 | 3.4 | 0.00 | 3.1 |
|  | Integral membrane protein DUF6 containing protein | Os11g0136300 | 3.1 | 0.00 | 2.4 |
|  | ^2^lipid transfer protein 1.14 (OsLTP1.14) | Os11g0427800 | 2.8 | 0.00 | 1.9 |
|  | ^1,2^Arabinogalactan protein 24 (OsAGP24) | Os06g0318800 | 2.6 | 0.01 | 3.4 |
|  | NADP-malic enzyme (OscytME2) | Os05g0186300 | 2.4 | 0.00 | 2.2 |
|  | ^1,2^Arabinogalactan protein 3 | Os03g0188500 | 2.4 | 0.01 | 2.0 |
|  | cystathionine b-synthase domain containing protein (OsCBSX11) | Os02g0639300 | 2.4 | 0.03 | 2.2 |
|  | lipid transfer protein d6 (OsLTPd6) | Os04g0415800 | 2.3 | 0.01 | 1.1 |
|  | Physical impedance induced protein | Os03g0718800 | 2.3 | 0.01 | 2.3 |
|  | monosaccharide transporter 6 (OsMST6) | Os07g0559700 | 2.2 | 0.01 | 3.8 |
|  | ^1^ABC transporter superfamily ABCG subgroup member 1 (OsABCG1) | Os01g0121600 | 2.2 | 0.00 | 3.6 |
|  | ^1^VQ motif-containing protein 1 (OsVQ1) | Os01g0278000 | 2.2 | 0.02 | 3.3 |
|  | ^2^COBRA-like protein 4 | Os10g0497700 | 2.1 | 0.03 | 4.0 |
|  | Pectinesterase inhibitor domain containing protein | Os12g0283400 | 2.0 | 0.01 | 5.8 |
| Carbohydrate metabolic process | | | | | |
|  | ^1,2^Xyloglucan endo-1,4-beta-D-glucanase (OsXTH9) | Os04g0604300 | 3.1 | 0.00 | 3.3 |
|  | ^1,2^galactinol synthase 2 | Os07g0687900 | 3.3 | 0.00 | 5.3 |
|  | ^1,2^Galactinol synthase (Fragment) (OsGolS1) | Os03g0316200 | 3.6 | 0.00 | 4.8 |
|  | ^1,2^Raffinose synthase | Os01g0170000 | 2.8 | 0.00 | 2.7 |
|  | ^2^Beta-amylase PCT-BMYI (EC 3.2.1.2) | Os03g0141200 | 3.4 | 0.00 | 3.9 |
|  | Alpha-amylase isozyme 3D precursor | Os08g0473900 | 2.5 | 0.00 | 4.5 |
|  | Glycosyl transferase, family 8 protein | Os03g0678800 | 2.7 | 0.04 | 3.8 |
|  | Glycosyl transferase, family 31 domain containing protein | Os11g0502700 | 2.6 | 0.00 | 3.0 |
|  | Xylanase inhibitor protein I precursor | Os06g0356800 | 2.3 | 0.00 | 3.4 |
| Plant hormone | | | | | |
|  | ^1^Chloroplast phytoene synthase 3. (phytoene synthase 3, OsPSY3) | Os09g0555500 | 2.0 | 0.00 | 6.9 |
|  | ^2^OsJAZ4, OsJAZ 10 | Os03g0181100 | 2.9 | 0.04 | 4.6 |
|  | ^1,2^OsJAZ3, OsJAZ 9 | Os03g0180800 | 3.9 | 0.01 | 5.3 |
|  | GIBBERELLIN 2-OXIDASE 9 (OsGA2ox9) | Os02g0630300 | 4.0 | 0.00 | 5.8 |
|  | Gibberellin regulated protein family protein | Os07g0592000 | 2.3 | 0.00 | 1.7 |
| ROS detoxification | | | | | |
|  | ^1^Senescence-associated protein 12 | Os03g0241900 | 2.6 | 0.01 | 2.9 |
|  | Nudix hydrolase 18 | Os06g0255400 | 2.7 | 0.03 | 3.0 |
|  | ^1^Mss4-like domain containing protein | Os05g0126800 | 2.5 | 0.00 | 2.4 |
|  | ankyrin protein kinase-like | Os04g0218400 | 2.2 | 0.00 | 1.6 |
|  | prx130, class III peroxidase 130 | Os11g0112400 | 2.4 | 0.00 | 3.5 |

Superscripts 1-4 on references reports observations of the following:

1. Twenty genes in the co-regulatory network of *OsERF48*

2. Genes which expression was confirmed through RT-PCR

**Table S2.** Nodes (genes) constituting the *OsERF48* co-regulatory network.

| **Locus ID** | **Description** | **Transcription Factor** | **Gene Symbol** |
| --- | --- | --- | --- |
| **Os01g0121600** | ***OsABCG1*** |  |  |
| **Os01g0135700** | ***OsCML16*** |  |  |
| **Os01g0170000** | ***RS5*** |  |  |
| Os01g0186900 | Conserved hypothetical protein. |  |  |
| Os01g0205900 | Similar to Class III peroxidase GvPx2b (Fragment). |  |  |
| **Os01g0278000** | ***OsVQ1*** |  |  |
| Os01g0498300 | Protein of unknown function DUF563 family protein. |  |  |
| Os01g0513400 | Protein of unknown function DUF789 family protein. |  |  |
| Os01g0583100 | Protein phosphatase 2C family protein. |  |  |
| Os01g0672400 | Drought induced 19 family protein. |  |  |
| Os01g0699600 | Protein kinase-like domain containing protein. |  |  |
| **Os01g0797600** | ***AP37*** | AP2-EREBP | ERF3 |
| Os01g0846300 | Similar to Protein phosphatase 2C. |  |  |
| Os01g0858200 | Similar to DEAH (Asp-Glu-Ala-His) box polypeptide 16. |  |  |
| Os01g0955100 | Similar to Avr9/Cf-9 rapidly elicited protein 57 (Fragment). |  |  |
| Os02g0532900 | Glycoside hydrolase, family 17 protein. |  |  |
| Os02g0558500 | Conserved hypothetical protein. |  |  |
| Os02g0566400 | Conserved hypothetical protein. |  |  |
| **Os02g0669100** | ***OsLEA23/DIP1*** |  |  |
| Os02g0677300 | Similar to CRT/DRE binding factor 1. | AP2-EREBP | DREB1G |
| Os02g0682300 | Zinc finger, RING-type domain containing protein. |  |  |
| Os02g0687200 | Protein of unknown function DUF581 family protein. |  |  |
| Os02g0696500 | Concanavalin A-like lectin/glucanase domain containing protein. |  |  |
| Os02g0702100 | Conserved hypothetical protein. |  |  |
| Os02g0733900 | Conserved hypothetical protein. |  |  |
| **Os02g0764700** | ***OsDERF5*** | AP2-EREBP |  |
| Os02g0766700 | Similar to Abscisic acid responsive elements-binding factor (ABA-responsive element binding protein 2) (AREB2). | bZIP |  |
| Os02g0789600 | Similar to DEM2. |  |  |
| Os03g0107700 | Similar to EL2 protein. |  | EL2 |
| Os03g0125100 | Similar to Beta-ring hydroxylase (Fragment). |  |  |
| Os03g0152000 | Heavy metal transport/detoxification protein domain containing protein. |  |  |
| **Os03g0180800** | ***OsJAZ3*** |  | JAZ3 |
| **Os03g0188500** | **OsAGP3** |  |  |
| Os03g0191900 | Pathogenesis-related transcriptional factor and ERF domain containing protein. | AP2-EREBP |  |
| Os03g0197100 | Similar to Sugar transporter protein. |  |  |
| **Os03g0241900** | ***OsSAP12*** |  |  |
| Os03g0247900 | Amino acid-binding ACT domain containing protein. |  | ACR9 |
| Os03g0292100 | Protein phosphatase 2C family protein. |  |  |
| Os03g0301200 | Similar to COBRA-like protein 7 precursor. |  |  |
| **Os03g0316200** | ***OsGolS1*** |  |  |
| Os03g0351300 | Glycoside hydrolase, family 14 protein. |  |  |
| Os03g0353400 | Similar to Poly(A)-binding protein C-terminal interacting protein 6. |  |  |
| Os03g0640000 | Patatin family protein. |  |  |
| Os03g0645900 | Carotenoid oxygenase family protein. |  | NCED1 |
| Os03g0656500 | Similar to K-exchanger-like protein. |  |  |
| Os03g0820300 | Similar to ZPT2-14. | C2H2 |  |
| Os03g0820400 | Similar to ZPT2-13. | C2H2 |  |
| Os04g0414500 | Conserved hypothetical protein. |  |  |
| Os04g0461600 | Similar to Fw2.2. |  |  |
| Os04g0518400 | Similar to Phenylalanine ammonia-lyase (Fragment). |  |  |
| Os04g0531100 | C2 calcium/lipid-binding region, CaLB domain containing protein. |  | RPP16 |
| Os04g0572400 | Similar to CRT/DRE binding factor 1. | AP2-EREBP | DREB1E |
| **Os04g0604300** | ***OsXTH9*** |  |  |
| **Os04g0610400** | ***OsAP2-39*** | AP2-EREBP |  |
| Os04g0669200 | Similar to Ethylene response factor 2 (Ethylene response factor 3). | AP2-EREBP |  |
| Os04g0674000 | Conserved hypothetical protein. |  |  |
| Os05g0102600 | Zinc finger, FYVE/PHD-type domain containing protein. |  |  |
| **Os05g0126800** | ***Mss4-like*** |  |  |
| Os05g0217000 | Protein of unknown function DUF1070 family protein. |  |  |
| Os05g0380900 | Similar to Polcalcin Jun o 2 (Calcium-binding pollen allergen Jun o 2). |  |  |
| Os05g0427200 | Glycosyl transferase, family 31 protein. |  |  |
| Os05g0457200 | Similar to Protein phpsphatase 2C (PP2C) (EC 3.1.3.16). |  |  |
| Os05g0497200 | Similar to Ethylene-responsive transcription factor 11 (Ethylene-responsive element binding factor 11) (EREBP-11) (AtERF11). | AP2-EREBP |  |
| Os05g0497300 | Similar to Ethylene response factor 2. | AP2-EREBP |  |
| Os05g0516700 | Conserved hypothetical protein. |  |  |
| Os05g0537400 | Similar to Protein phosphatase 2C. |  |  |
| Os05g0545300 | Protein kinase-like domain containing protein. |  |  |
| Os05g0545400 | Protein kinase-like domain containing protein. |  |  |
| **Os06g0127100** | ***OsDREB1c*** | AP2-EREBP | DREB1C |
| Os06g0133400 | Conserved hypothetical protein. |  |  |
| Os06g0133500 | Conserved hypothetical protein. |  |  |
| Os06g0203600 | Conserved hypothetical protein. |  |  |
| **Os06g0318800** | ***OsAGP24*** |  |  |
| **Os06g0683400** | ***OsCCD1*** |  |  |
| Os07g0245100 | Similar to Cytosine deaminase (EC 3.5.4.1). |  |  |
| Os07g0587500 | Armadillo-like helical domain containing protein. |  |  |
| Os07g0589600 | Conserved hypothetical protein. |  |  |
| Os07g0602900 | Protein of unknown function DUF1675 family protein. |  |  |
| **Os07g0687900** | ***OsLEA24/OsGolS2*** |  |  |
| Os08g0205700 | Non-protein coding transcript, unclassifiable transcript. |  |  |
| Os08g0293100 | Conserved hypothetical protein. |  |  |
| **Os08g0408500** | ***OsERF48*** | AP2-EREBP |  |
| Os08g0490100 | Similar to PBF protein. | C2C2-Dof |  |
| Os08g0524100 | Similar to Type II inositol-1,4,5-trisphosphate 5-phosphatase 12 (EC 3.1.3.36) (At5PTase12) (FRAGILE FIBER3 protein). |  |  |
| Os09g0378700 | U box domain containing protein. |  |  |
| Os09g0385700 | Zinc finger, AN1-type domain containing protein. |  |  |
| Os09g0522000 | Similar to CBF-like protein. | AP2-EREBP | DREB1B |
| **Os09g0555500** | ***OsPSY3*** |  |  |
| Os10g0391400 | ZIM domain containing protein. |  |  |
| Os10g0580900 | Conserved hypothetical protein. |  |  |
| Os11g0498600 | Similar to HVA22 protein. |  |  |
| Os12g0242500 | Conserved hypothetical protein. |  |  |
| Os12g0594000 | WD40-like domain containing protein. |  |  |

Bold indicate 20 genes involved in DEGs of *ROX^OsERF48^* root.

**Table S3.** Primers used in this study.

|  | Name | 5' to 3' |
| --- | --- | --- |
| **OsERF48:GFP** | |  |
|  | pHBT_B_inf_OsERF48_F | TTGCTCCGTGGATCC tcg atg gca gct gct ata gaa gg |
|  | pHBT_inf_OsERF48_N | AAAGCGGCCGCAAATgttattgttgttgagcagcgagtc |
|  | pHBT_inf_sGFP_N | ATT TGC GGC CGC TTT Atggtgagcaagggcgagga |
|  | pHBT_P_sGFP_R | TTGAACGATCTGCAGttacttgtacagctcgtccatgc |
| **OsNF-YA7-mcherry** | |  |
|  | pHBT_B_inf_OsNF-YA7_F | TTGCTCCGTGGATCC atg aag cca gat ggt gaa act cag c |
|  | pHBT_inf_OsNF-YA7_N | AAAGCGGCCGCAAATtacaacatcggacgcatctgcac |
|  | pHBT_inf_mChry_N | ATT TGC GGC CGC TTT ATGGTGAGCAAGGGCGAGGAG |
|  | pHBT_P_mCherry_R | TTGAACGATCTGCAGCTACTTGTACAGCTCGTCCATGC |
| **Overexpression** | |  |
|  | OsERF48_N_F | gcggccgcATGGCAGCTGCTATAGAAG |
|  | OsERF48_Sm_R | cccgggCTAGTTATTGTTGTTGAGCAGCG |
|  | OsERF48_RNAi_F | AAA AAG CAG GCT TTA TAC CCC CCT TGT AAA GT |
|  | OsERF48_RNAi_R | AGAAAGCTGGGTATGTAGTGGAACCCGTGGAC |
|  | OsERF49_tag_F | Ggatcc ATGGCAGCTGCTATAGAAGGAA |
|  | OsERF49_tag_R | Gcggccgc ATTATTGTTGTTGAGCAGCG |
| **Transactivation assay in yeast** | |  |
|  | pGBKT7-R1-OsERF48_1_F | ATG GAG GCC GAA TTC Tcgatggcagctgctatagaagg |
|  | pGBKT7-R1-OsERF48_39_F | ATG GAG GCC GAA TTC Aacttctcctcggccggagt |
|  | pGBKT7-R1-OsERF48_85_F | ATG GAG GCC GAA TTC Atgaagcccgcttcggcg |
|  | pGBKT7-B-OsERF48_162-R | C AGG TCG ACG GAT CCcgttgtccgggaagttgagc |
|  | pGBKT7-B-OsERF48_220-R | C AGG TCG ACG GAT CCagtggtcggagccggagga |
|  | pGBKT7-B-OsERF48_280_R | C AGG TCG ACG GAT Ccagttattgttgttgagcagcgagtc |
|  | **primer combination for OsERF48 deletion mutant for transactication assay (Figure 1D)** | |
|  | pGBKT7-R1-OsERF48_1_F | OsERF48F |
|  | pGBKT7-B-OsERF48_280_R |  |
|  | pGBKT7-R1-OsERF48_39_F | OsERF48Δc4 |
|  | pGBKT7-B-OsERF48_280_R |  |
|  | pGBKT7-R1-OsERF48_85_F | OsERF48Δc3c4 |
|  | pGBKT7-B-OsERF48_280_R |  |
|  | pGBKT7-R1-OsERF48_1_F | OsERF48Δc1c2 |
|  | pGBKT7-B-OsERF48_162-R |  |
|  | pGBKT7-R1-OsERF48_1_F | OsERF48Δc1 |
|  | pGBKT7-B-OsERF48_220-R |  |
| **Transient expression assay in protoplast** | |  |
|  | **Effector** |  |
|  | pHBT-B-OsERF48_1_F | TTG CTC CGT GGA TCC Tcgatggcagctgctatagaagg |
|  | pHBT-N-OsERF48_220-R | CCT ATC TGC GGC CGC gtggtcggagccggagga |
|  | pHBT-N-OsERF48_280_R | CCT ATC TGC GGC CGC gttattgttgttgagcagcgagtcc |
|  | **primer combination for OsERF48 deletion mutant for protoplast transient expression assay (Figure 7I)** | |
|  | pHBT-B-OsERF48_1_F | OsERF48F |
|  | pHBT-N-OsERF48_280_R |  |
|  | pHBT-B-OsERF48_1_F | OsERF48Δc1 |
|  | pGBKT7-B-OsERF48_220-R |  |
|  | **Reporter** |  |
|  | OsCML16_pro_Fw | GCC AGT GCC AAG CTT tttgctgggctggcactatt |
|  | OsCML16_pro_Rv | TGG CGT CTT CCA TGG agaagtgtggctgtggtgag |
| **ChIP q-PCR** | |  |
|  | OsCML16_P1_F | actccctcatctccgaaaaccg |
|  | OsCML16_P1_F | agggcttgaagacacgactc |
|  | OsCML16_P2_F | acatgcaagaggctgattcgg |
|  | OsCML16_P2_F | aggacctcgacgggtggttg |
|  | OsCML16_P3_F | cgctgctcgagccgattgaa |
|  | OsCML16_P3_R | gcttaggtgccatctaagtggc |
|  | OsCML16_P4_F | ccatacatgtgcaccccctt |
|  | OsCML16_P4_R | ggcctttccctgctgcatag |
|  | OsDREB1c_P1_F | atgggttcgttgggacttgg |
|  | OsDREB1c_P1_F | gtacggactacggagacgga |
|  | OsDREB1c_P2_F | aaaagcttgcctttgtcgcc |
|  | OsDREB1c_P2_F | gtacttcgctaggtgtcggg |
|  | OsDREB1c_P3_F | gacatctccagccaattccag |
|  | OsDREB1c_P3_R | aaggggatggagaggaagaagt |
|  | OsLEA24_P1_F | caaacgttgagcccgcaacac |
|  | OsLEA24_P1_F | cacttgtacagcgtcggcag |
|  | OsLEA24_P2_F | tatcgccggctctagcaccag |
|  | OsLEA24_P2_F | agccggcgttaattccatcc |
|  | OsLEA24_P3_F | gcaaaacggttggtgcgtta |
|  | OsLEA24_P3_R | gctgtggtgatgttcgcttg |
|  | Os02g0771600_P1_F | caaaccctatccggtcgcct |
|  | Os02g0771600_P1_F | agccatcggaggagagaagag |
|  | Os02g0771600_P2_F | aagagcgagccatcacagac |
|  | Os02g0771600_P2_F | gccaacctcctctaaaggct |
|  | Os02g0771600_P3_F | gcctataaattgggcgctcg |
|  | Os02g0771600_P3_R | gttgatcaccgggaaggagg |
| **qRT_PCR** | |  |
|  | OsCML16_RT_F | Ctgacaaggccaagacggag |
|  | OsCML16_RT_R | Cggcgctgctaattacacat |
|  | OsDREB1c_RT_F | Actgatgatcgcgagttggag |
|  | OsDREB1c_RT_R | Gcatgctgtccctcgtagtag |
|  | OsSAP12_RT_F | Gaactcgaaacctgcccaga |
|  | OsSAP12_RT_R | Attgccctttgggtcgagag |
|  | OsCCD1_RT_F | Gtcctcatggtcaggctcag |
|  | OsCCD1_RT_R | ggaatccgcgaaccaatgag |
|  | DIP1_RT_F | Gcttaattggggcgtgtgtg |
|  | DIP1_RT_R | Taccaaggcagaatgcccag |
|  | OsLEA24_RT_F | tcgaagccatggaggttcac |
|  | OsLEA24_RT_R | agcactccttaataagcgcca |
|  | OsGolS1_RT_F | CAAGCCGATCCCACTGATCT |
|  | OsGolS1_RT_R | TGTCCATGTTCGCCTCCTTG |
|  | RS5_RT_F | Ccaggatgcggagttcaagt |
|  | RS5_RT_R | CTGGGGCTTTGCTGAAGAGA |
|  | OsAGP3_RT_F | GGATGGATGATTTGATCGACGC |
|  | OsAGP3_RT_R | TCATCTCATCAAACATGACAAGTG |
|  | OsXTH9_RT_F | GCAAAGAGCAGCAGTTCCAC |
|  | OsXTH9_RT_R | TTGTCCGCGTGGTTCTTCAT |
|  | OsJAZ3_RT_F | Tcgattgatcgccatgtggt |
|  | OsJAZ3_RT_R | Cgccgcgataactagggtaa |
|  | OsAP2-39_RT_F | Tcgtagaaaagtctagagcgacg |
|  | OsAP2-39_RT_R | Aaggaactgtaacaccggga |
